# Ubiquitin-SUMO Circuitry Controls Activated Fanconi Anemia ID Complex Dosage in Response to DNA Damage

**DOI:** 10.1016/j.molcel.2014.12.001

**Published:** 2015-01-08

**Authors:** Ian Gibbs-Seymour, Yasuyoshi Oka, Eeson Rajendra, Brian T. Weinert, Lori A. Passmore, Ketan J. Patel, Jesper V. Olsen, Chunaram Choudhary, Simon Bekker-Jensen, Niels Mailand

**Affiliations:** 1 Ubiquitin Signaling Group, The Novo Nordisk Foundation Center for Protein Research, Faculty of Health and Medical Sciences, University of Copenhagen, 2200 Copenhagen, Denmark; 2 MRC Laboratory of Molecular Biology, Francis Crick Avenue, Cambridge CB2 0QH, UK; 3 Department of Proteomics, The Novo Nordisk Foundation Center for Protein Research, Faculty of Health and Medical Sciences, University of Copenhagen, 2200 Copenhagen, Denmark

## Abstract

We show that central components of the Fanconi anemia (FA) DNA repair pathway, the tumor suppressor proteins FANCI and FANCD2 (the ID complex), are SUMOylated in response to replication fork stalling. The ID complex is SUMOylated in a manner that depends on the ATR kinase, the FA ubiquitin ligase core complex, and the SUMO E3 ligases PIAS1/PIAS4 and is antagonized by the SUMO protease SENP6. SUMOylation of the ID complex drives substrate selectivity by triggering its polyubiquitylation by the SUMO-targeted ubiquitin ligase RNF4 to promote its removal from sites of DNA damage via the DVC1-p97 ubiquitin segregase complex. Deregulation of ID complex SUMOylation compromises cell survival following replication stress. Our results uncover a regulatory role for SUMOylation in the FA pathway, and we propose that ubiquitin-SUMO signaling circuitry is a mechanism that contributes to the balance of activated ID complex dosage at sites of DNA damage.

## Highlights

• The Fanconi anemia ID complex (FANCI/FANCD2) is SUMOylated after DNA damage
• ID complex SUMOylation is regulated by ATR, the FA core complex, PIAS1/4, and SENP6
• SUMO-dependent ubiquitylation by RNF4 allows ID complex removal from DNA by DVC1/p97
• Deregulated ID complex SUMOylation compromises cell survival following DNA damage

## Graphical Abstract

**Figure:**
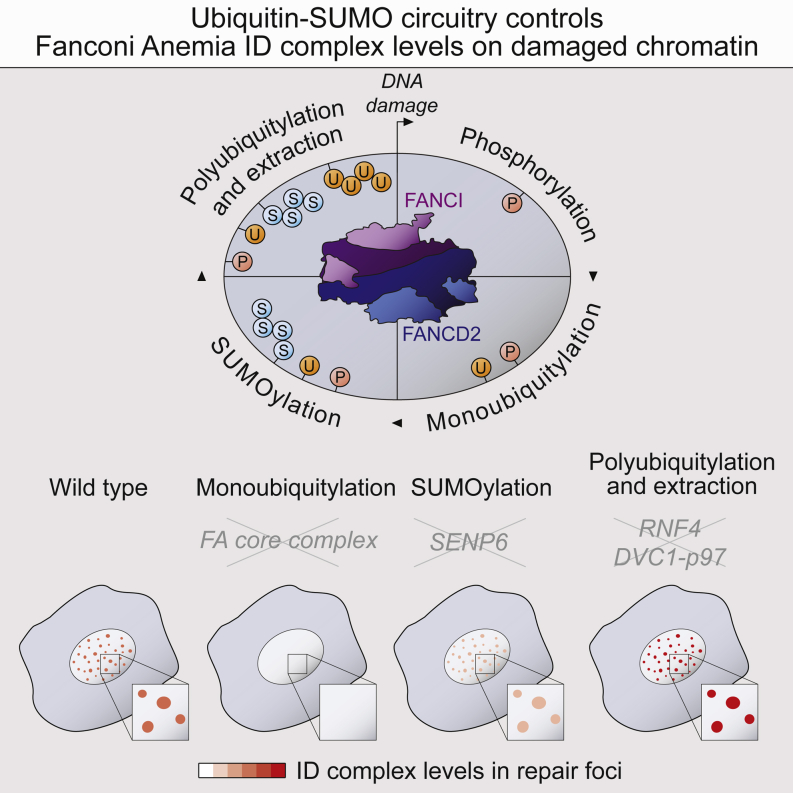

## Teaser

Gibbs-Seymour et al. describe how multiple posttranslational modifications (phosphorylation, ubiquitylation, and SUMOylation) cooperate to regulate the chromatin retention of the Fanconi anemia ID complex, a central component of the Fanconi anemia tumor suppressor pathway, at sites of DNA damage.

## Introduction

Cellular genomes are under incessant attack from genotoxic insults, which elicit a protective cellular mechanism termed the DNA damage response (DDR) (Jackson and Bartek, 2009). The DDR includes a diverse set of signal transduction pathways that act to sense different types of DNA lesions and effectively repair the damage to minimize genomic instability that might be propagated to daughter cells (Ciccia and Elledge, 2010). Posttranslational modifications (PTMs) of proteins are one major mechanism to regulate the DDR. Both ubiquitin- and SUMO-dependent signaling play key roles in various genome maintenance pathways, modulating individual protein function to facilitate the numerous activities and protein interactions required in DNA repair (Jackson and Durocher, 2013; Mailand et al., 2013). The ubiquitylation and SUMOylation status of target substrates is fine-tuned by the presence of deubiquitylating enzymes (DUBs) or SUMO proteases, respectively, which may reverse and/or edit the modifications to create a dynamic signaling mechanism (Hickey et al., 2012; Komander et al., 2009).

Crosstalk between ubiquitin and SUMO exists at multiple levels and functions to integrate various signaling cues (Jackson and Durocher, 2013). For instance, polySUMO2 chains may be recognized by a class of E3 ubiquitin ligases termed SUMO-targeted ubiquitin ligases (STUbLs), which interact noncovalently with SUMO-modified target proteins through SUMO-interacting motifs (SIMs) to facilitate the formation of ubiquitin chains of various linkages on these substrates (Poulsen et al., 2013; Tatham et al., 2008). Thus, in this manner, SUMOylation can drive ubiquitylation of target proteins. Depending on the ubiquitin chain type, STUbL activity may serve to recruit proteins with ubiquitin-binding domains or may promote protein degradation. As an example of the latter, the STUbL RNF4 ubiquitylates SUMOylated MDC1 and RPA in the response to DNA double-strand breaks (DSBs), regulating their proteasome-dependent turnover at DNA lesions (Galanty et al., 2012; Vyas et al., 2013; Yin et al., 2012). However, despite its importance, the full extent of this ubiquitin-SUMO crosstalk in genome maintenance pathways is not known.

Fanconi anemia (FA) is a rare disorder resulting from bialleic mutations in at least 16 different gene products (FANCA-FANCQ) (Kottemann and Smogorzewska, 2013). The clinical manifestation of inactivating mutations in these genes includes congenital abnormalities, failure of the bone marrow, and cancer predisposition (Crossan and Patel, 2012). FA patient cells exhibit increased chromosomal aberrations and a striking sensitivity to agents that cause DNA interstrand crosslinks (ICLs) (Kee and D’Andrea, 2012). ICLs are one of the most cytotoxic lesions that threaten genome integrity, posing a physical obstruction to ongoing DNA replication and transcription machineries (Kim and D’Andrea, 2012; Kottemann and Smogorzewska, 2013). The repair of ICLs is a hazardous cellular endeavor because the decision to activate the FA pathway leads to the programmed formation of a DSB, which, if repaired erroneously, can lead to a loss of genetic material and/or genomic rearrangements (Adamo et al., 2010; Pace et al., 2010). The FA pathway is therefore subject to strict regulation by PTMs, and the FANCI/FANCD2 complex (ID complex) is the epitome of such regulation. FANCI is phosphorylated by ATR/ATM, which has been proposed to stabilize the interaction between FANCD2 and FANCI (Ishiai et al., 2008; Joo et al., 2011). FANCI phosphorylation is a requisite step for the subsequent site-specific monoubiquitylation on FANCD2 at K561 and FANCI on K523, carried out by the FA core complex, a large multisubunit ubiquitin ligase (Kim and D’Andrea, 2012). These monoubiquitylations function to license the ID complex, facilitating recruitment of nucleases such as XPF/ERCC1, which are responsible for mediating incisions proximal to the ICL, unhooking the crosslink with the concomitant formation of a DSB (Hodskinson et al., 2014; Klein Douwel et al., 2014; Knipscheer et al., 2009). The FA pathway uses translesion synthesis, homologous recombination, and nucleotide excision repair to complete the repair process (Knipscheer et al., 2009; Zhang and Walter, 2014) (Figure S1A available online). ID complex monoubiquitylation is antagonized by the USP1-UAF1 DUB complex (Cohn et al., 2007). Deletion of *USP1* in chicken DT40 cells or in mouse models leads to enhanced chromatin loading of the ID complex in the absence of exogenous DNA damage, although the levels of chromatin loaded monoubiquitylated FANCD2 are similar after mitomycin C (MMC) treatment (Kim et al., 2009; Oestergaard et al., 2007; Rajendra et al., 2014). This suggests that there might be other mechanisms of regulating the levels of chromatin loaded ID complex at DNA lesions.

Relatively few targets of DNA damage-dependent SUMOylation have been reported in human cells. Here, we found that FANCI and FANCD2 are dynamically regulated by SUMOylation in response to genotoxic stress. ID complex SUMOylation potentiates polyubiquitylation by RNF4, which in turn promotes ID complex chromatin extraction via the DVC1-p97 ubiquitin segregase complex. We propose that this ubiquitin-SUMO signaling functions to control the balance of activated ID complex dosage at sites of DNA damage.

## Results

### FANCI and FANCD2 Are SUMOylated in Response to DNA Damage

**Figure 1:**
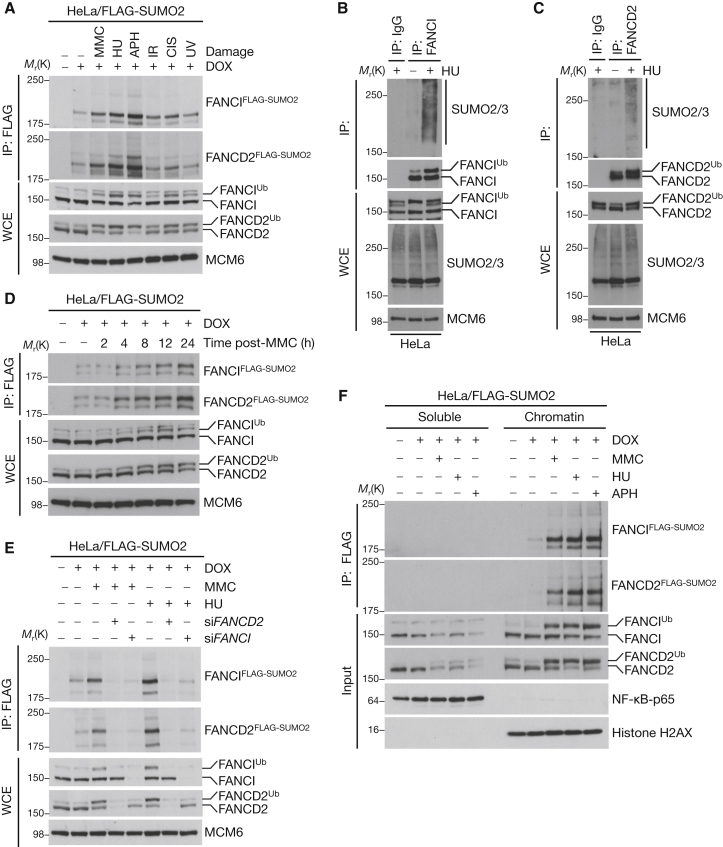
The FANCI/FANCD2 (ID) Complex Is SUMOylated after DNA Damage (A) Stable HeLa/FLAG-SUMO2 cells were treated with doxycycline (DOX) for 24 hr to induce FLAG-SUMO2 expression. Cells were then lysed under denaturing conditions, subjected to FLAG immunoprecipitation (IP), and analyzed by immunoblotting using the indicated antibodies. WCE, whole-cell extract; CIS, cisplatin. MCM6 is used as a loading control. (B) HeLa cells were treated with HU for 24 hr and lysed under denaturing conditions, before IP with anti-FANCI antibody or preimmune serum (IgG). Immunopurified material was analyzed by immunoblotting with the indicated antibodies. (C) Same as (B) except FANCD2 antibody was used for the IP. (D) HeLa/FLAG-SUMO1 cells were induced with DOX and then subjected to MMC for various durations and processed as in (A). (E) HeLa/FLAG-SUMO2 cells treated with FANCI or FANCD2 siRNA and induced with DOX were subjected to MMC or HU for a further 24 hr and processed as in (A). (F) HeLa/FLAG-SUMO2 cells were biochemically fractionated, diluted in denaturing buffer, and processed as in (A). See also Figure S1.

In a proteomic screen for SUMOylation targets in response to DNA damage, we identified FANCI as exhibiting increased SUMOylation after ionizing radiation (IR) and ultraviolet light (UV) (data not shown). While independent studies have identified FANCI peptides in proteomic screens for SUMO targets (Golebiowski et al., 2009; Tatham et al., 2011; Yin et al., 2012), there has been no previously reported functional role for SUMOylation of factors in the FA pathway and we therefore decided to investigate this further. For these analyses, we used inducible HeLa/His-FLAG-SUMO1 or SUMO2 cell lines in which overexpression of the SUMO transgenes was limited to less than 2-fold the level of endogenous SUMO (Figures S1B and S1C) (Danielsen et al., 2012). We confirmed that both FANCI as well as its binding partner in the ID complex, FANCD2, are targeted for DNA damage-induced modification by wild-type (WT) but not conjugation-deficient (ΔGG) SUMO1 and SUMO2 in response to a range of genotoxic stresses, most prominently after treatment with agents that cause replication fork stalling, including MMC, hydroxyurea (HU), and aphidicolin (APH) (Figure 1A; Figures S1D and S1E). We validated the physiological relevance of these findings by showing that both endogenous FANCI and FANCD2 are modified by endogenous SUMO2/3 in a DNA damage-inducible manner (Figures 1B and 1C). In time courses, the extent of ID complex SUMOylation correlated with its monoubiquitylation level in response to both MMC and HU, suggesting that these modifications are linked (Figures 1D and S1F). Similar to monoubiquitylation, ID complex SUMOylation was strictly dependent on the presence of either protein and occurred exclusively on chromatin (Figures 1E, 1F, and S1G–S1I). We conclude that the ID complex, a key component of the FA pathway, becomes SUMOylated in response to replication fork stalling.

### ATR, FA Core Complex, and PIAS1/PIAS4-Dependent ID Complex SUMOylation

**Figure 2:**
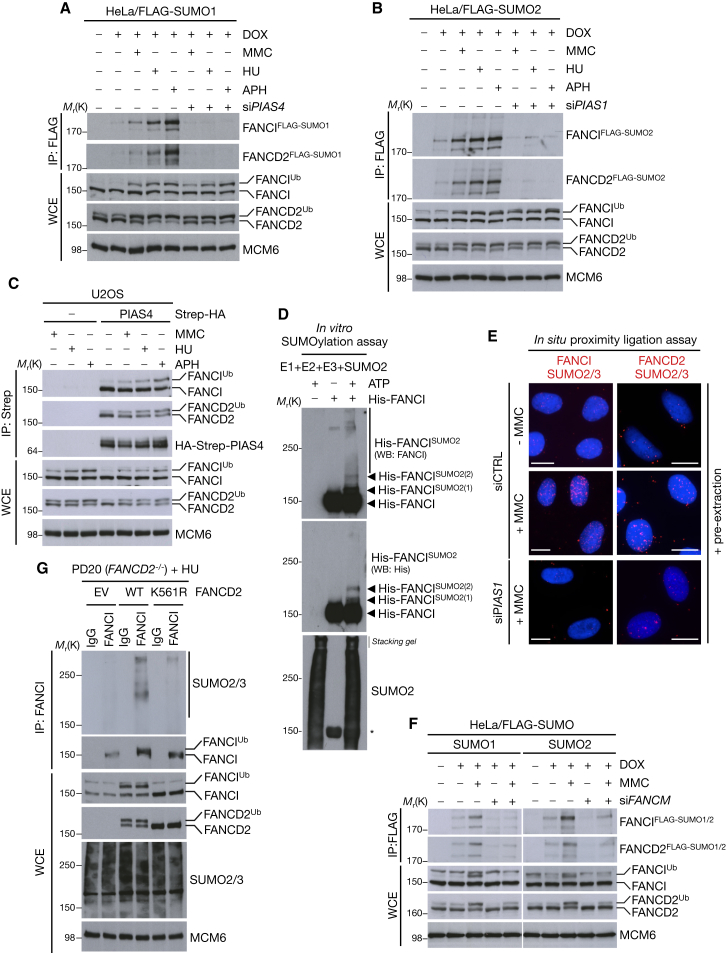
ID complex SUMOylation Requires ATR, the FA Core Complex, and the SUMO E3 Ligases PIAS1 and PIAS4 (A) HeLa/FLAG-SUMO1 cells induced with DOX were transfected with siRNA against the SUMO E3 ligase PIAS4 and then subjected to MMC, HU, or APH for a further 24 hr. Cells were then lysed under denaturing conditions, subjected to FLAG IP, and analyzed by immunoblotting using the indicated antibodies. (B) Same as (A), using HeLa/FLAG-SUMO2 cells and PIAS1 siRNA. (C) U2OS cells transfected with Strep-HA-PIAS4 or empty vector (−) were subjected to replication stress using indicated agents for 24 hr. Strep-HA-PIAS4 complexes were purified with Strep-Tactin Sepharose and analyzed by immunoblotting. (D) Recombinant His-FANCI was SUMOylated in vitro and analyzed by immunoblotting with anti-His and anti-FANCI antibodies. ^∗^, denotes unmodified His-FANCI band after reblotting. (E) U2OS cells transfected with control (CTRL) or PIAS1 siRNA were treated with MMC, pre-extracted, and then subjected to in situ proximity ligation assay using the indicated combination of antibodies. Scale bar represents 10 μm. (F) HeLa/FLAG-SUMO1/2 cells induced with DOX were transfected with FANCM siRNA and subjected to MMC for 24 hr. Cells were then processed as in (A). (G) FANCD2-deficient human fibroblasts (PD20) complemented with empty vector (EV), FANCD2 WT, or K561R mutant were treated with HU for 24 hr before lysis under denaturing conditions. FANCD2 was immunopurified using anti-FANCD2 antibody. Bound material was analyzed by immunoblotting with indicated antibodies. See also Figure S2.

Having established the spatio-temporal dynamics of ID complex SUMOylation, we then characterized the underlying enzymatic machinery. Knockdown of the SUMO E2 enzyme UBC9 impaired SUMOylation of the ID complex, as expected (data not shown). Next, we performed a small-scale siRNA screen against known SUMO E3s and found that PIAS1 and PIAS4 are required for ID complex SUMO1 and SUMO2 modification, respectively, but not for FA pathway activation in response to replication stress (Figures 2A, 2B, and S2A–S2D). We also observed the ID complex in immunoprecipitates of PIAS1 and PIAS4 (Figures 2C and S2E). We then sought to reconstitute the entire SUMOylation reaction in vitro, using full-length recombinant His-tagged FANCI (Rajendra et al., 2014). SUMO2 modification of FANCI was enhanced in the presence of PIAS1, consistent with our in vivo data (Figure 2D). We further visualized PIAS1-dependent, MMC-stimulated ID complex SUMOylation under endogenous conditions by the in situ proximity ligation assay, using a combination of FANCI or FANCD2 antibodies together with a SUMO2/3 antibody (Figures 2E and S2F). Together, these results indicate that two SUMO E3 ligases, PIAS1 and PIAS4, promote DNA damage-dependent SUMOylation of the chromatin loaded ID complex.

Because ID complex SUMOylation occurs exclusively on chromatin, we reasoned that the phosphorylation and monoubiquitylation events required for loading the complex onto chromatin might be required for its SUMOylation. Indeed, we found that depletion of ATR suppressed the DNA damage-dependent SUMOylation of the ID complex (Figure S2G). Likewise, individual depletion of the FA core complex components FANCM, FANCA, or FANCL severely reduced ID complex SUMOylation (Figures 2F, S2H, and S2I). Finally, using *FANCD2−/−* cells (PD20) and derivative lines reconstituted with FANCD2 WT or a monoubiquitylation-deficient K561R mutant (Garcia-Higuera et al., 2001), we established directly that SUMOylation of FANCI is strongly dependent on FANCD2 monoubiquitylation (Figure 2G).

### SENP6 Antagonizes PIAS-Mediated FANCD2 and FANCI SUMOylation

**Figure 3:**
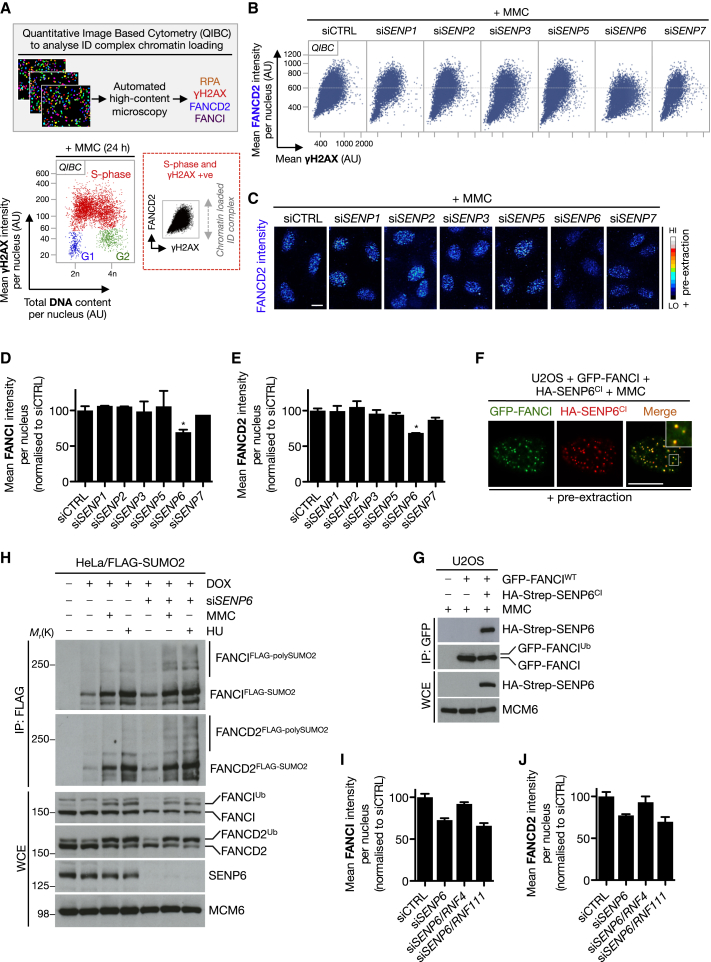
SENP6 Antagonizes ID Complex SUMOylation (A) Schematic of QIBC methodology used to analyze ID complex chromatin loading. (B) U2OS cells were transfected with the indicated siRNAs against known SUMO proteases, treated with MMC (0.3 μM) for 24 hr, and then pre-extracted in situ to isolate chromatin bound proteins. Immunostained cells were processed for QIBC as outlined in (A). (C) Examples from (B) of FANCD2 chromatin-bound levels using QIBC. Scale bar represents 10 μm. (D) Quantification of mean FANCI chromatin-bound intensity using the same approach as (B). Data represent mean ± SEM from two independent experiments. ^∗^p < 0.05. (E) Same as (D), but using FANCD2 antibody. (F) U2OS cells cotransfected with GFP-FANCI and HA-SENP6^CI^ were treated with MMC (0.3 μM), fixed 4 hr later, and immunostained with HA antibody. Scale bar represents 10 μm. (G) Same as (F) except cells were subjected to GFP IP followed by immunoblotting with indicated antibodies. (H) HeLa/FLAG-SUMO2 cells induced with DOX were treated with SENP6 siRNA and subjected to MMC or HU for 24 hr. Cell lysates were subjected to FLAG IP under denaturing conditions before immunoblotting with indicated antibodies. (I) U2OS cells transfected with indicated siRNAs were treated with MMC and processed for QIBC. Cells were stained with FANCI antibody and chromatin-bound FANCI was quantified. Data represent mean ± SEM from two independent experiments. (J) Same as (I) but using FANCD2 antibody. See also Figure S3.

We next investigated whether SUMOylation of the ID complex would be antagonized by one or more SUMO proteases (SENPs). To this end, we individually depleted cells of known SENPs and determined ID complex chromatin loading after MMC treatment by means of quantitative image-based cytometry (QIBC) (Toledo et al., 2013) (Figure 3A). By combining ID complex immunostaining with a number of DNA damage markers (e.g., RPA and γH2AX), QIBC allows for the discrimination of S phase cells, which in turn facilitates quantitative spatio-temporal analysis of ID complex loading in response to replication stress in nonsynchronized populations (Figures S3A–S3E). Using QIBC, we found that depletion of SENP6 caused decreased ID complex chromatin retention after MMC treatment, when most cells are in S/G2 phase (Figures 3A–3E and S3F). Based on this finding, we reasoned that SENP6 might localize to sites of DNA damage. We did not observe stable accumulation of SENP6 at sites of laser microirradiation (Figure S3G); however, when coexpressed with FANCI, a catalytically inactive SENP6 mutant (SENP6^CI^) became resistant to pre-extraction and fully colocalized with GFP-FANCI in foci after MMC (Figure 3F). This finding was supported biochemically by coimmunoprecipitation of the two proteins (Figure 3G). In addition, depletion of SENP6 led to the appearance of more prominent polySUMO2 chains on the ID complex (Figure 3H), further suggesting that SENP6 is a functional regulator of the ID complex.

We reasoned that in the absence of SENP6, the increased polySUMO2 chains on the ID complex might activate a STUbL, which in turn would promote their subsequent polyubiquitylation and removal, accounting for the decreased chromatin retention of the ID complex in response to DNA damage. To test this, we codepleted either of the two known human STUbLs, RNF4 and RNF111, both of which have been implicated in genome maintenance pathways (Galanty et al., 2012; Poulsen et al., 2013; Yin et al., 2012), together with SENP6 and assessed ID complex retention after MMC by QIBC. Depletion of SENP6 together with RNF4, but not RNF111, rescued chromatin-bound ID complex levels (Figures 3I and 3J), suggesting that in the absence of SENP6, RNF4 corrupts the FA pathway. SUMOylation might thus be a mechanism to regulate activated ID complex at sites of DNA damage and we therefore sought to investigate the role of RNF4 in the FA pathway.

### RNF4 Regulates the FA Pathway by Limiting Activated ID Complex Dosage at DNA Lesions

**Figure 4:**
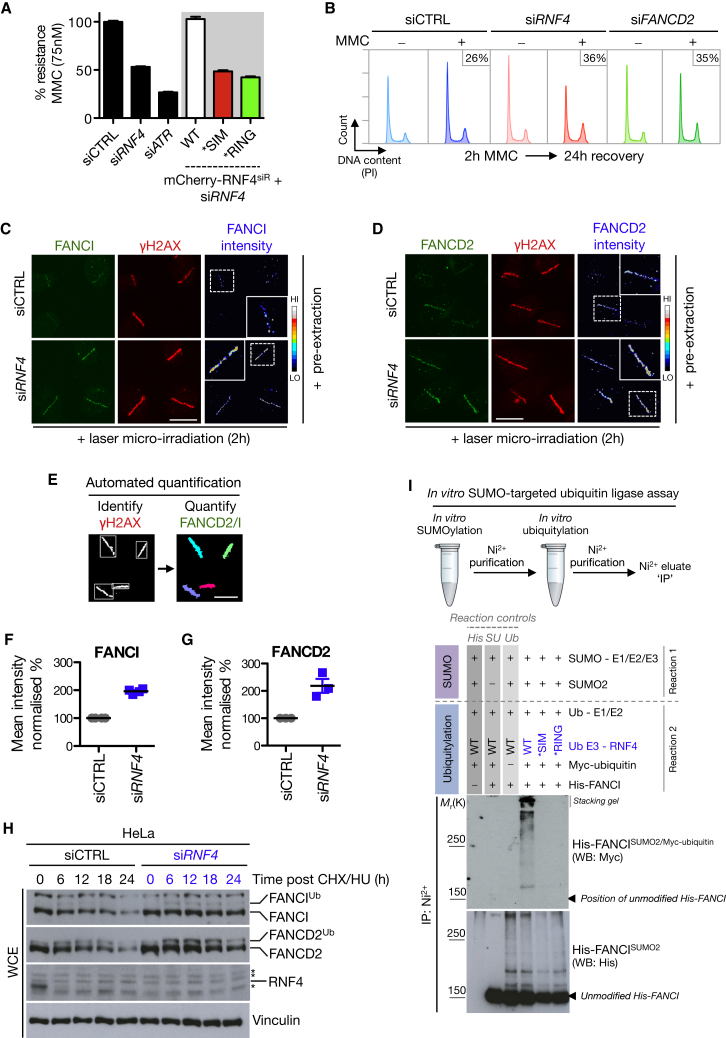
RNF4 Is a Regulator of the FA Pathway and Polyubiquitylates the SUMOylated ID Complex (A) Cellular fitness of U2OS cells transfected with control (CTRL), ATR, or RNF4 siRNA was assessed using the multicolor competition assay (MCA) (Smogorzewska et al., 2007). Stable U2OS/mCherry-RNF4 siRNA-resistant (si^R^) cell lines were used for RNF4 allele complementation analysis. Data represent mean ± SEM from three independent experiments (B) HeLa cells were transfected with the indicated siRNAs, exposed to a pulse of MMC (50 ng/ml) for 2 hr, and then allowed to recover for 24 hr before fixation and analysis by flow cytometry. The proportion of cells in G2/M phase is indicated. (C) U2OS cells transfected with control (CTRL) or RNF4 siRNA were subjected to laser microirradiation, pre-extracted and fixed 2 hr later, and immunostained with FANCDI and γH2AX antibodies. Scale bar represents 10 μm. (D) Same as (C) except with FANCD2 antibody. (E) QIBC strategy to analyze data generated from laser microirradiation experiments in an automated unbiased manner. (F) Quantification of normalized mean FANCI intensities at sites of laser microirradiation. Each data point represents the quantification of 75–150 cells from three independent experiments. (G) Same as (F) but for FANCD2. (H) HeLa cells transfected with control (CTRL) or RNF4 siRNA were treated with cycloheximide (CHX) and HU for the indicated times. Protein extracts were analyzed by immunoblotting with indicated antibodies. ^∗^, denotes cross-reactive bands. (I) His-FANCI was SUMOylated in vitro as in Figure 2D, purified on Ni^2+^ agarose and then subjected to in vitro ubiquitylation by RNF4. The Ni^2+^ beads were then washed extensively and analyzed by immunoblotting with indicated antibodies. See also Figure S4.

We established a cellular model system to deplete endogenous RNF4 and complement cells with mCherry-tagged siRNA-resistant alleles of WT RNF4 or mutant forms containing inactivating point mutations in either its SIM or RING domain (denoted ^∗^SIM and ^∗^RING, respectively) (Figures S4A–S4D) (Tatham et al., 2008). Assessment of cellular fitness using the multicolor competition assay revealed that RNF4 depletion sensitized cells to MMC and that RNF4 WT, but neither the ^∗^SIM nor the ^∗^RING mutant was able to complement RNF4 depletion (Figures 4A and S4E). Notably, the ^∗^RING mutant of RNF4 colocalized with FANCD2 at sites of stalled forks, which we could not detect for RNF4 WT and ^∗^SIM, suggesting that RNF4 WT only interacts transiently with stalled forks and the ^∗^RING mutant acts as a substrate trap (Figure S4F). Supporting this, RNF4 ^∗^RING efficiently immunoprecipitated the ID complex (Figure S4G). A feature of FA patient cells is enhanced G2/M arrest after ICL-inducing clastogens, indicative of a failure to adequately repair damage incurred during the prior S phase (Akkari et al., 2001). We found that both FANCD2 and RNF4 depletion gave rise to such a phenotype after low dose MMC treatment (Figure 4B). Based on the known role for RNF4 in targeting proteins for removal from DSB sites, we surmised that ID complex SUMOylation might potentiate a similar mechanism. Indeed, both FANCI and FANCD2 accumulated to supraphysiological levels at sites of DNA damage in RNF4 depleted cells (Figures 4C and 4D). Using QIBC, we found that ID complex levels were increased 2-fold at DNA lesions (Figures 4E–4G), suggesting that RNF4 negatively regulates ID complex retention at sites of DNA damage.

Prompted by these findings, we tested whether ID complex SUMOylation might trigger its RNF4-dependent ubiquitylation, using multiple approaches. Endogenous FANCI/FANCD2 could be polyubiquitylated in an RNF4-dependent manner (Figure S4H). RNF4 can catalyze the formation of both K48- and K63-linked ubiquitin chains in vitro (Tatham et al., 2008). Consistently, using ubiquitin mutant expression constructs, we found that endogenous FANCD2 and FANCI could be modified by both K48- and K63-linked ubiquitin chains after replication stress (Figures S4I and S4J). Depletion of RNF4 also reduced the SUMO-ubiquitin conjugates covalently attached to the ID complex (Figure S4K). Based on these findings, we investigated if RNF4 regulates ID complex stability using a cycloheximide chase approach. Depletion of RNF4 compromised ID complex degradation, suggesting that at least one function of RNF4-dependent polyubiquitylation is to target the ID complex for proteasomal destruction (Figure 4H). To directly assess whether SUMOylated FANCI is a target for RNF4-dependent ubiquitylation, recombinant His-FANCI was subjected to an in vitro STUbL assay, using WT or mutant forms of recombinant RNF4 (Figure S4L). This revealed that while WT RNF4 effectively ubiquitylated SUMOylated but not unmodified FANCI, the ^∗^SIM and ^∗^RING mutants showed no such activity (Figure 4I). Collectively, these data strongly suggest that RNF4 polyubiquitylates the SUMOylated ID complex to limit its DNA damage-induced chromatin loading.

### The DVC1-p97 Ubiquitin-Selective Segregase Complex Promotes Extraction of the ID Complex from Damaged Chromatin

**Figure 5:**
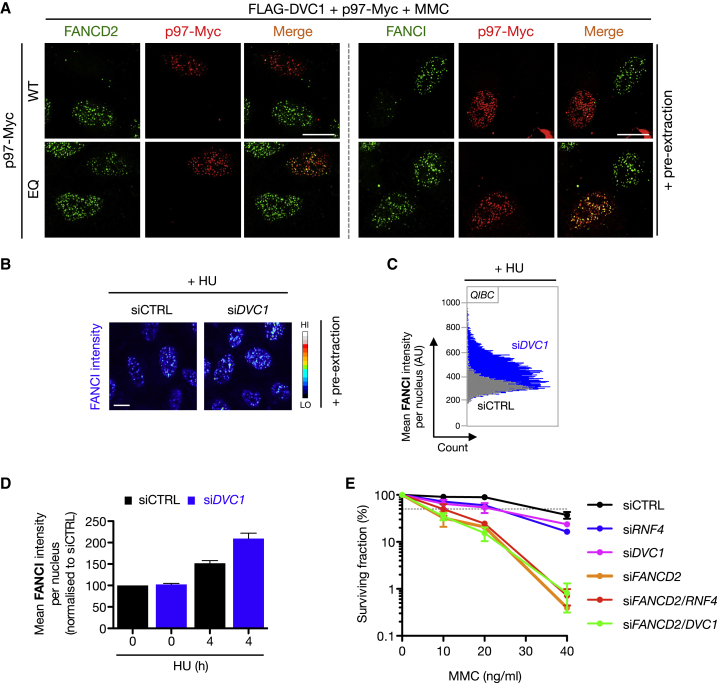
The DVC1-p97 Complex Promotes Extraction of the ID Complex from Sites of DNA Damage (A) U2OS cells cotransfected with FLAG-DVC1 and p97-Myc WT or ATPase-dead (EQ) were treated with MMC (0.3 μM) for 24 hr and then pre-extracted, fixed, and immunostained with indicated antibodies. Scale bar represents 10 μm. (B) U2OS cells were transfected with control (CTRL) or DVC1 siRNA, treated with HU for 24 hr, pre-extracted, fixed, immunostained, and analyzed by QIBC. A representative image is shown for chromatin-bound FANCI. (C) Representative plot from QIBC analysis from (B). (D) Quantification of data from (B). Data represent mean ± SEM from two biologically independent experiments. (E) Colony formation assay using HeLa cells transfected with indicated siRNAs and subjected to various doses of MMC for 24 hr. Data represent mean ± SEM from two independent experiments using technical triplicates per datapoint. See also Figure S5.

We hypothesized that the RNF4-mediated polyubiquitylation of the SUMOylated ID complex might trigger its active removal from chromatin, potentially mediated by the DVC1-p97 complex, which we have previously shown promotes ubiquitin-dependent extraction of proteins from stalled replication forks (Davis et al., 2012; Mosbech et al., 2012). Indeed, co-overexpression of DVC1 with WT, but not ATPase-dead (EQ), p97 led to the removal or significant reduction of FANCD2 and FANCI from MMC- and HU-induced stalled forks (Figures 5A and S5A). The ability of DVC1 to promote extraction of the ID complex from stalled forks depends on both the interaction with p97 and with ubiquitylated target proteins via its SHP-box and UBZ domains, respectively (Figure S5B). Depletion of FANCA or FANCD2 had no effect on DVC1 accumulation at MMC- and HU-induced stalled forks, suggesting that ID complex monoubiquitylation is not required for DVC1 recruitment to stalled forks (Figure S5C). All of the above observations were specific to DVC1 because no other p97 adaptor we tested could elicit the same phenotype (Figure S5D). Using QIBC, we found that depletion of endogenous DVC1 led to increased chromatin loading of the ID complex after HU or MMC treatments, suggesting that like RNF4, DVC1 tempers ID complex chromatin loading in response to replication stress (Figures 5B–5D; data not shown). Consistently, knockdown of RNF4 or DVC1 sensitized cells to MMC to the same extent, and codepletion of RNF4 and FANCD2 or DVC1 and FANCD2 led to a similar MMC sensitivity as knockdown of FANCD2 alone (Figures 5E and S5E), suggesting an epistatic relationship between these proteins and the FA pathway. Thus, whereas DVC1 and RNF4 both have functions outside ICL repair, depletion of these regulators does not give rise to enhanced MMC sensitivity in the absence of FANCD2 because their roles in this context are likely dependent on ID complex-mediated activities to provide a substrate for their function. Collectively, these results suggest that the DVC1-p97 ubiquitin segregase complex functions to limit activated ID complex dosage on chromatin in a SUMO- and ubiquitin-dependent manner.

### FANCI SUMOylation Regulates Activated ID Complex Dosage at Sites of DNA Damage

**Figure 6:**
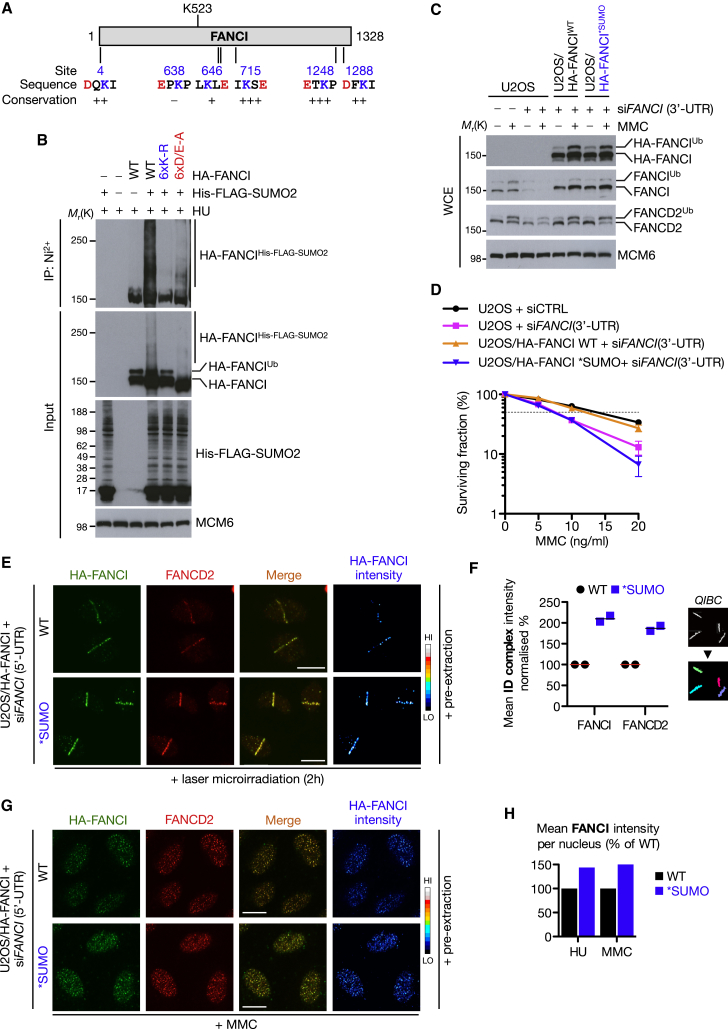
FANCI SUMOylation Regulates Activated ID Complex Dosage at Sites of DNA Damage (A) Location and conservation of potential SUMOylation sites in FANCI. Modified lysine residues in consensus SUMOylation motifs are shown in blue with the acidic residue in red. (B) HeLa cells transfected with HA-FANCI wild-type (WT) or HA-FANCI SUMO-site mutants (6xK-R or 6xD/E-A) together with His-FLAG-SUMO2 were subjected to HU treatment for 24 hr. SUMO conjugates were purified under denaturing conditions using Ni^2+^ agarose and analyzed by immunoblotting with HA antibody. (C) Indicated cell lines transfected with control or FANCI (3′UTR) siRNA were treated with MMC for 24 hr and whole-cell extracts were analyzed by immunoblotting with indicated antibodies. (D) Clonogenic survival of indicated U2OS cell lines depleted of endogenous FANCI using the 3′-UTR siRNA where indicated and treated with various doses of MMC. Data represent mean ± SEM from three independent experiments using technical triplicates per datapoint. (E) U2OS/HA-FANCI WT or ^∗^SUMO cells transfected with FANCI (5′-UTR) siRNA were subjected to laser microirradiation, pre-extracted, and fixed after 2 hr and then immunostained with HA and FANCD2 antibodies. Scale bar represents 10 μm. (F) QIBC analysis of normalized mean HA-FANCI and FANCD2 intensities from (E). Data represent mean ± SEM from two independent experiments. (G) U2OS/HA-FANCI WT or ^∗^SUMO cells transfected with FANCI (5′-UTR) siRNA were treated with MMC (0.3 μM) for 24 hr and immunostained as in (E). Scale bar represents 10 μm. (H) U2OS/HA-FANCI cell lines treated as in (G) or with HU were analyzed by QIBC. Mean intensity of chromatin loaded HA-FANCI was normalized to HA-FANCI WT. See also Figure S6.

To characterize the specific effects of FANCI and FANCD2 SUMOylation, we next sought to produce a SUMO-deficient mutant of one or both proteins. Mass spectrometry analysis of in vitro SUMOylated FANCI (Figure 2D) revealed a single modified lysine site, K715, which is one of six potential SUMO sites identified by in silico analysis (Figures 6A and S6A; data not shown). However, single mutation of this lysine had no impact on FANCI SUMO2 modification and it required progressive loss of all six consensus SUMO sites to completely abrogate FANCI SUMOylation (Figures 6B, S6B, and S6C). Importantly, this FANCI ^∗^SUMO mutant was fully functional, because it was monoubiquitylated as efficiently as FANCI WT (Figure 6B). Mutation of the acidic amino acids in the SUMO modification consensus sequences (6xD/E-A) also strongly reduced FANCI SUMOylation (Figures 6A and 6B), similarly to what has been reported for other SUMOylated proteins (Hendriks et al., 2014). The six FANCI SUMO sites are clustered in three regions in FANCI (Figures S6A and S6D) and, with the possible exception of K646, all SUMO sites are surface accessible once viewed within the context of the ID complex crystal structure (Figure S6D).

Having produced a SUMOylation-deficient FANCI mutant, we analyzed its impact on key determinants of FA pathway efficacy. Cell lines were created expressing either WT or SUMOylation-deficient (^∗^SUMO) HA-tagged FANCI alleles and endogenous FANCI was depleted using siRNA targeting the 3′-UTR (Figure 6C). Interestingly, using these cell lines, we found that SUMOylation of endogenous FANCD2 was strongly suppressed in cells expressing the FANCI ^∗^SUMO mutant (Figure S6E), indicating that abrogation of FANCI SUMOylation suppresses overall ID complex SUMOylation. While FANCI WT could complement the sensitivity to MMC resulting from depletion of endogenous FANCI, the ^∗^SUMO mutant failed to promote such rescue (Figure 6D), despite FANCD2 monoubiquitylation, and thus FA pathway activation, was not compromised in these cells (Figure 6C). We reasoned that cellular sensitivity might result if the ^∗^SUMO mutant phenocopied RNF4 depletion by dysregulating levels of activated ID complex at DNA lesions, thus potentially interfering with the timely recruitment of downstream repair factors. Indeed, the FANCI ^∗^SUMO mutant exhibited enhanced accumulation at DNA damage sites, also promoting enhanced FANCD2 accumulation at the lesions (Figures 6E–6H). In line with this, expression of FANCI ^∗^SUMO increased the endogenous burden of DNA damage (data not shown), possibly contributing to the inability of this mutant to rescue the MMC sensitivity of cells lacking endogenous FANCI (Figure 6D). Together, our data suggest that FANCI SUMOylation is important to regulate the dosage of activated ID complex on chromatin, and an abrogation of this regulation promotes genome instability.

### SUMOylation-Deficient FANCI Is Refractory to Regulation by RNF4, SENP6, and DVC1

**Figure 7:**
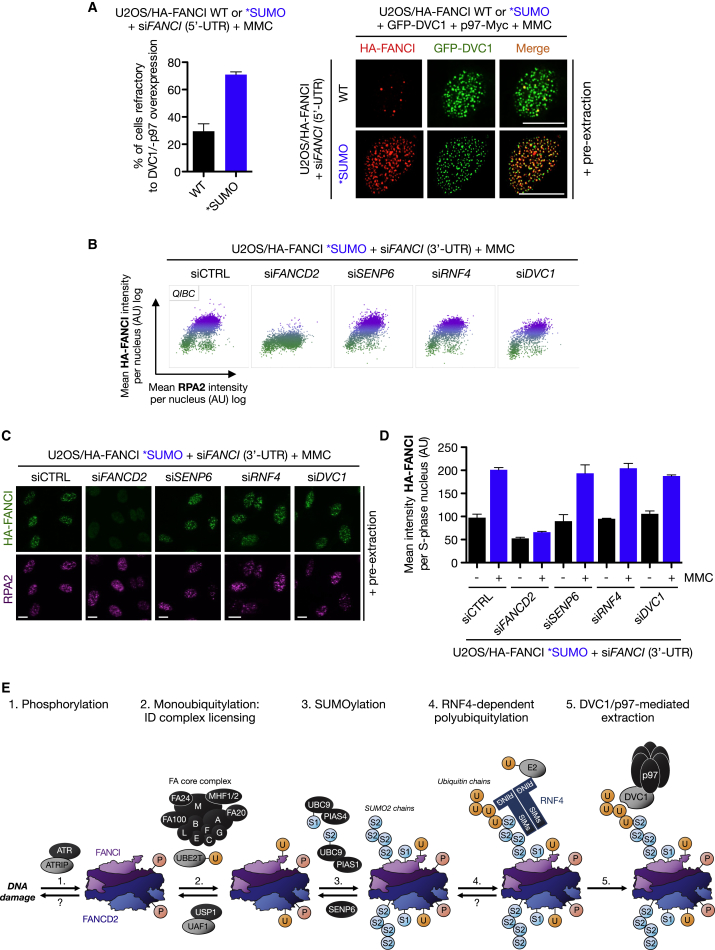
SUMOylation-Deficient FANCI Is Insensitive to Regulation by RNF4, SENP6, and DVC1 (A) U2OS/HA-FANCI WT or ^∗^SUMO cells transfected with FANCI (5′-UTR) siRNA were transfected with GFP-DVC1 and p97-Myc plasmids and then treated with MMC (0.3 μM) for 4 hr, pre-extracted, fixed, and immunostained with HA antibody. Scale bar represents 10 μm. Data represent mean ± SEM from two independent experiments. For each data point, 200–400 cells were quantified. (B) U2OS/HA-FANCI ^∗^SUMO cells were depleted of endogenous FANCI together with the indicated factors and processed for QIBC. (C) Representative images from data shown in (B). (D) Quantification of QIBC data from (B). Data represent mean ± SEM from two independent experiments. (E) Model of ubiquitin-SUMO circuitry in regulation of the ID complex (see Discussion for details). Modification locations do not represent actual sites. Question marks denote potential, but presently unknown, mechanism of regulation. P, phosphorylation; U, ubiquitin; S1, SUMO1; and S2, SUMO2. See also Figure S7.

The SUMOylation-deficient FANCI mutant allowed us to revisit all of our prior conclusions and test whether the observed phenotypes could be attributed to the SUMOylated lysine residues. First, to assess if the FANCI SUMOylation sites were important for DVC1-p97-dependent extraction, we co-overexpressed DVC1 and p97 in FANCI WT and ^∗^SUMO cells depleted of endogenous FANCI. We found that the FANCI ^∗^SUMO mutant was largely refractory to DVC1-p97-mediated removal, whereas FANCI WT could be effectively extracted at DNA lesions (Figure 7A). Extending these findings, we predicted that the chromatin loading of the FANCI ^∗^SUMO mutant would also be refractory to modulation by SENP6 and RNF4. Indeed, while depletion of FANCD2 inhibited loading of both FANCI WT and ^∗^SUMO as expected, depletion of SENP6, RNF4 and DVC1 only impacted on the chromatin loading of FANCI WT but not the ^∗^SUMO mutant, implying that these lysine residues in FANCI channel the SUMO-dependent regulation of the ID complex on chromatin (Figures 7B–7D and S7). At least some of the FANCI SUMO sites have been found to be also modified by ubiquitin (Kim et al., 2011; Wagner et al., 2011). While we cannot rule out that an inability to ubiquitylate these residues may affect FANCI ^∗^SUMO mutant phenotypes, our collective data strongly suggest that SUMOylation of these sites is the main determinant for ID complex removal from damaged chromatin.

## Discussion

The ID complex is regulated by the sequential actions of phosphorylation and monoubiquitylation, which serves as a central activating step within the FA pathway to promote ubiquitin-dependent recruitment of downstream nucleases. However, beyond these PTMs it is not clear if the ID complex is subject to additional levels of regulation. Here, we provide substantial evidence that SUMOylation of the ID complex integrates various posttranslational signaling cues to modulate its effective dosage at DNA lesions. Mechanistically, this occurs through a series of exquisitely regulated steps (Figure 7E). The concerted actions of phosphorylation and monoubiquitylation function to license the ID complex for nuclease recruitment and promote its chromatin loading. PIAS1/4-mediated SUMOylation of the chromatin loaded ID complex potentiates recognition and subsequent polyubiquitylation by RNF4. The SUMO-dependent polyubiquitin chains on the ID complex then provide a substrate for the DVC1-p97 ubiquitin segregase complex to promote extraction of the ID complex from chromatin. Thus, multiple PTMs act to fine-tune the balance of activated ID complex at DNA lesions.

An obvious question arising from our study is: why should there be such an elaborate mechanism to control ID complex dosage? The simplest explanation is that it helps limit nuclease-mediated incisions in the DNA. Another likely possibility is that it facilitates differential responses of the ID complex during the dynamic process of DNA repair. As has been noted for many other SUMO-modified proteins, the SUMOylated form of the ID complex represents only a small fraction of the total cellular pool. Therefore, only this small fraction will be subject to the regulation described here, and each step in this circuitry provides further channeling of selectivity. Moreover, within the model that we propose, each PTM on the ID complex can be removed or dynamically modified by further modes of regulation, some of which are presently unknown (Figure 7E). In this regard, because RNF4 is activated by SUMO2 chains (Rojas-Fernandez et al., 2014), the antagonistic actions of PIAS1 and SENP6 may be key determinants of ID complex chromatin loading and SUMO2 chain length of the ID complex might be a rate-limiting step in promoting its extraction via RNF4-DVC1-p97.

ID complex SUMOylation was most strongly enhanced upon stresses that cause replication fork stalling, for example, HU, MMC, and APH. The common undercurrent to these stresses is the production of single-stranded DNA, which was previously shown to be a potent signaling cue for protein SUMOylation (Psakhye and Jentsch, 2012). This study also proposed the concept of “protein group synergy,” which posits that multiple proteins within signaling pathways are SUMOylated, and the overall efficacy of the specific DNA repair pathway is only affected by the concomitant loss of many of these SUMO-driven events (Psakhye and Jentsch, 2012). While SUMO-dependent regulation of the FA pathway might, in principle, also be an example of such “protein group synergy,” the results presented here suggest that site-specific SUMOylation of the ID complex is important to limit activated ID complex dosage, rather than the concerted actions of multiple SUMO-modified proteins.

The monoubiquitylation licensing of the ID complex is antagonized by the DUB complex USP1/UAF1; however, it is currently unknown how PTMs regulate USP1/UAF1 after DNA damage. Unregulated activity of USP1 during ICL repair thus risks inhibiting the efficacy of the FA pathway, and as such does not provide selectivity to ID complex chromatin loading. We note that chromatin loading of the ID complex is not an effective marker for efficient ICL repair, supported by genetic evidence showing that concomitant loss of *USP1*/*FANCC* in chicken DT40 cells does not rescue cellular sensitivity to MMC even though FANCD2 monoubiquitylation is restored (Rajendra et al., 2014). In contrast, our data suggest that SUMOylation-driven polyubiquitylation of the ID complex provides a very selective means by which to regulate a minor fraction of chromatin loaded ID complex that does not risk affecting the bulk of the activated complex.

Budding yeast p97 (known as Cdc48) has been recently shown to be able to recognize and extract proteins from chromatin via cooperative binding to SUMO and ubiquitin (Bergink et al., 2013; Nie et al., 2012). The data reported here provide a link between DVC1-p97 and SUMO-dependent RNF4-mediated polyubiquitin chain formation in human cells, suggesting a clear and rational link between SUMO- and ubiquitin-dependent signaling in the negative regulation of protein complexes at DNA lesions. To what extent this RNF4-DVC1-p97 cooperativity is a general mechanism remains to be determined. Although we show that at least one function of the SUMO-dependent polyubiquitylation targets the ID complex for proteasomal degradation, we note, as others have (Howlett et al., 2009), that the ID complex has a long half-life after cycloheximide addition and not all p97 substrates are destined for degradation (Dantuma and Hoppe, 2012; Ndoja et al., 2014), which leads to the possibility that the ID complex could be recycled by the concerted actions of SUMO proteases and DUBs.

In summary, we define a ubiquitin- and SUMO-dependent signaling circuitry that acts to selectively limit ID complex dosage at DNA lesions. Furthermore, we propose that RNF4-DVC1-p97 signaling might be a general mechanism of integrating ubiquitin- and SUMO-dependent signaling to negatively regulate protein complexes at replication-associated DNA lesions to promote genome stability.

## Experimental Procedures

### Cell Culture

Human U2OS, HEK293T, and HeLa cells were cultured in Dulbecco's modified Eagle's medium containing 10% fetal bovine serum. HeLa cell lines expressing His_6_-FLAG-SUMO, U2OS/GFP-DVC1 cells, and U2OS/Strep-HA-ubiquitin cells were described previously (Danielsen et al., 2011, 2012; Mosbech et al., 2012). To generate stable U2OS/mCherry-RNF4 siRNA-resistant clones, U2OS cells were cotransfected with mCherry-RNF4^siR^ constructs together with pBabe-Puro and selected with puromycin, as described (Mosbech et al., 2012). Stable U2OS/HA-FANCI WT/^∗^SUMO cells were selected in puromycin after transfection with pIRESpuro3-HA-FANCI (a kind gift from Dr Tony Huang). FANCD2-deficient human fibroblasts (PD20), and derivative lines reconstituted with FANCD2 WT or FANCD2 K561R (Garcia-Higuera et al., 2001) were kind gifts from Alan D’Andrea (Dana Farber Cancer Institute).

### Immunochemical Methods

Isolation of SUMO and ubiquitin conjugates under denaturing conditions was performed as described (Damgaard et al., 2012; Danielsen et al., 2012). Purification of His_6_-FLAG-SUMO conjugates using Ni^2+^ agarose (QIAGEN) was performed exactly as described (Tatham et al., 2009). Purification of endogenous FANCI or FANCD2 for SUMO2/3 analysis was carried out essentially as described (Barysch et al., 2014). Chromatin fractionation and coimmunoprecipitation of protein complexes followed by immunoblotting analysis was performed as described previously (Mosbech et al., 2012).

### Multicolor Competition Assay, Colony Formation Assay, and Flow Cytometry

Cellular sensitivity to MMC was performed as described (Smogorzewska et al., 2007), using U2OS stable cell lines expressing either empty pAcGFP-C1 or pmCherry-C1 (both Clontech), which were generated previously (Gudjonsson et al., 2012) and kindly provided by Jiri Lukas (University of Copenhagen, Denmark), or in the case of rescue experiments, with the U2OS/mCherry-RNF4^siR^ cell lines. Cells were analyzed after 7 days using a flow cytometer (FACSCalibur, BD Biosciences). For colony formation assay, cells were treated with siRNA, plated at low densities, then treated with the indicated doses of MMC for 24 hr. Cells were then washed free of MMC and subsequently fixed and stained with crystal violet after 10–12 days. The surviving fraction at each dose was calculated after normalization to the plating efficiency of untreated samples. Cell cycle analysis was performed as described previously (Mosbech et al., 2012).

### Immunofluorescence, Laser Microirradiation, Microscopy, and QIBC

In nearly all instances, cells were pre-extracted with 0.2% Triton X-100/PBS for 3 min on ice, before fixation with 4% formaldehyde for 15 min. Cells were then subjected to another permeablilization step with 0.2% Triton X-100/PBS for 5 min and incubated with primary and secondary antibodies for 1–2 hr each. In situ proximity ligation assay was performed according to the manufacturer’s guidelines (Duolink). Confocal microscopy and laser microirradiation were performed exactly as described (Mosbech et al., 2012). Images were acquired under nonsaturating conditions for the sample exhibiting the highest signal intensity and the settings subsequently applied to all other samples. Fiji was used to create intensity profiles of acquired images. QIBC was performed exactly as described (Toledo et al., 2013).

### In Vitro SUMOylation STUbL Assays and Recombinant Protein Production

His-tagged chicken FANCI and FANCD2 were purified exactly as described previously (Rajendra et al., 2014). Recombinant His-Strep-HA-RNF4 was produced by the CPR Protein Production Unit, and the His-Strep-HA tag was removed by AcTEV protease (Invitrogen) according to the manufacturer’s instructions. For in vitro SUMOylation assays, components (SAE1/2, UBC9, PIAS1, SUMO2, FANCI, or FANCD2) were added to a total reaction volume of 30 μl in SUMOylation buffer (50 mM Tris [pH 7.5], 5 mM MgCl_2_, 0.6 mM dithiothreitol, 2 mM NaF) and incubated at 30°C for 2 hr. For STUbL assays, in vitro SUMOylation reactions scaled up 3-fold were diluted in 500 μl binding buffer (20 mM Tris [pH 7.5], 150 mM NaCl, 0.05% NP-40, 1 mM imidazole) and added to Ni^2+^ agarose for 2 hr at 4°C. Bound proteins were washed extensively in 50 mM Tris (pH 7.5), subjected to in vitro ubiquitylation by recombinant RNF4 at 37°C for 90 min, washed again, and analyzed with immunoblotting.

## Author Contributions

I.G.-S. performed all experiments, analyzed data, and cowrote the manuscript; Y.O. provided the HA-FANCI WT cell line; E.R. purified recombinant His-tagged FANCI and FANCD2; L.A.P. and K.J.P. cosupervised E.R.; B.T.W., C.C., and J.V.O. contributed mass spectrometry data; S.B.-J. cosupervised the project; N.M. conceived of original proteomic screen, supervised the project, analyzed data, and cowrote the manuscript.

## Supplemental Information

Document S1. Supplemental Experimental Procedures and Figures S1–S7

Document S2. Article plus Supplemental Information
