# Supplementary material for: Ubiquitin-SUMO Circuitry Controls Activated Fanconi Anemia ID Complex Dosage in Response to DNA Damage
Source: Mol Cell. 2015 Jan 8;57(1):150–64. doi: 10.1016/j.molcel.2014.12.001 (PMC4416315; doi:10.1016/j.molcel.2014.12.001)
Supplement: Document S1. Supplemental Experimental Procedures and Figures S1–S7 [file mmc1.pdf]

Molecular Cell, Volume *57*

Supplemental Information

Ubiquitin-SUMO Circuitry Controls Activated Fanconi Anemia ID Complex Dosage in Response to DNA Damage

Ian Gibbs-Seymour, Yasuyoshi Oka, Eeson Rajendra, Brian T. Weinert, Lori A. Passmore, Ketan J. Patel, Jesper V. Olsen, Chunaram Choudhary, Simon Bekker-Jensen, and Niels Mailand

Gibbs-Seymour et al., Figure S1

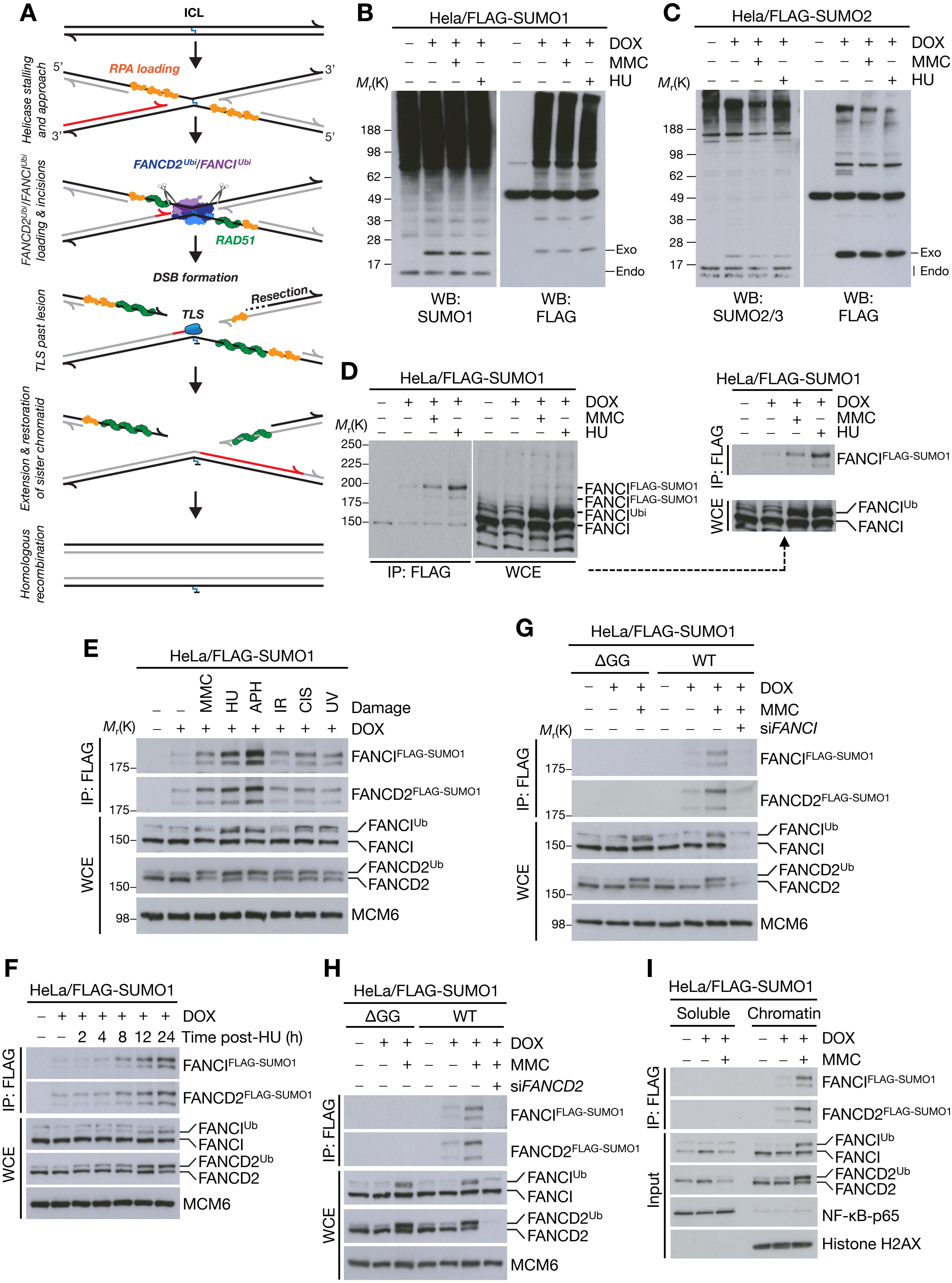

Figure S1 (related to Figure 1).
Endogenous ID complex is modified in response to DNA damage by the small ubiquitin-like modifiers SUMO1 and SUMO2.

- **A.** Current model of replication-dependent ICL repair (Knipscheer et al., 2009; Long et al., 2011; Raschle et al., 2008; Zhang and Walter, 2014).
- **B.** HeLa/FLAG-SUMO1 cells were treated with doxycycline (DOX) for 48 h to induce the expression of FLAG-SUMO1. Cells were subjected to mitomycin C (MMC) (1 µM) or hydroxyurea (HU) (2 mM) treatment for 24 h and protein extracts were analyzed by immunoblotting with the indicated antibodies. WCE, whole cell extracts.
- **C.** Same as (B) except with HeLa/FLAG-SUMO2 cells.
- **D.** Stable HeLa cell lines expressing wild type FLAG-SUMO2 were treated with doxycycline (DOX) for 24 h to induce FLAG-SUMO2 expression. Cells were then lysed under denaturing conditions, subjected to FLAG immunoprecipitation and then analysed by immunoblotting using the indicated antibodies. WCE, whole-cell extract; IP, immunoprecipitates. MCM6 is used as a loading control. *Left*, example of Western blot data, showing SUMO-modified forms of FANCI in the whole cell extract and after immunoprecipitation of the SUMO1. *Right*, same data but after cropping the important bands.
- **E.** HeLa/FLAG-SUMO2 cells were exposed to various genotoxic stresses and processed as in (D).
- **F.** HeLa/FLAG-SUMO1 cells were treated with HU (2 mM) for various durations before being processed as in (D).
- **G.** Stable HeLa cell lines expressing wild type (WT) or conjugation-deficient (ΔGG) FLAG-SUMO1 were transfected or not with FANCI siRNA and treated with doxycycline (DOX) for 24 h to induce FLAG-SUMO2 expression. Cells were then subjected to MMC treatment for an additional 24 h and collected. SUMOylation of FANCI and FANCD2 was analysed as in (D).
- **H.** Same as in (G), using FANCD2 siRNA.
- **I.** HeLa/FLAG-SUMO1 cells were biochemically fractionated, diluted in denaturing buffer before immunopurification and immunoblotting with the indicated antibodies.

Gibbs-Seymour et al., Figure S2

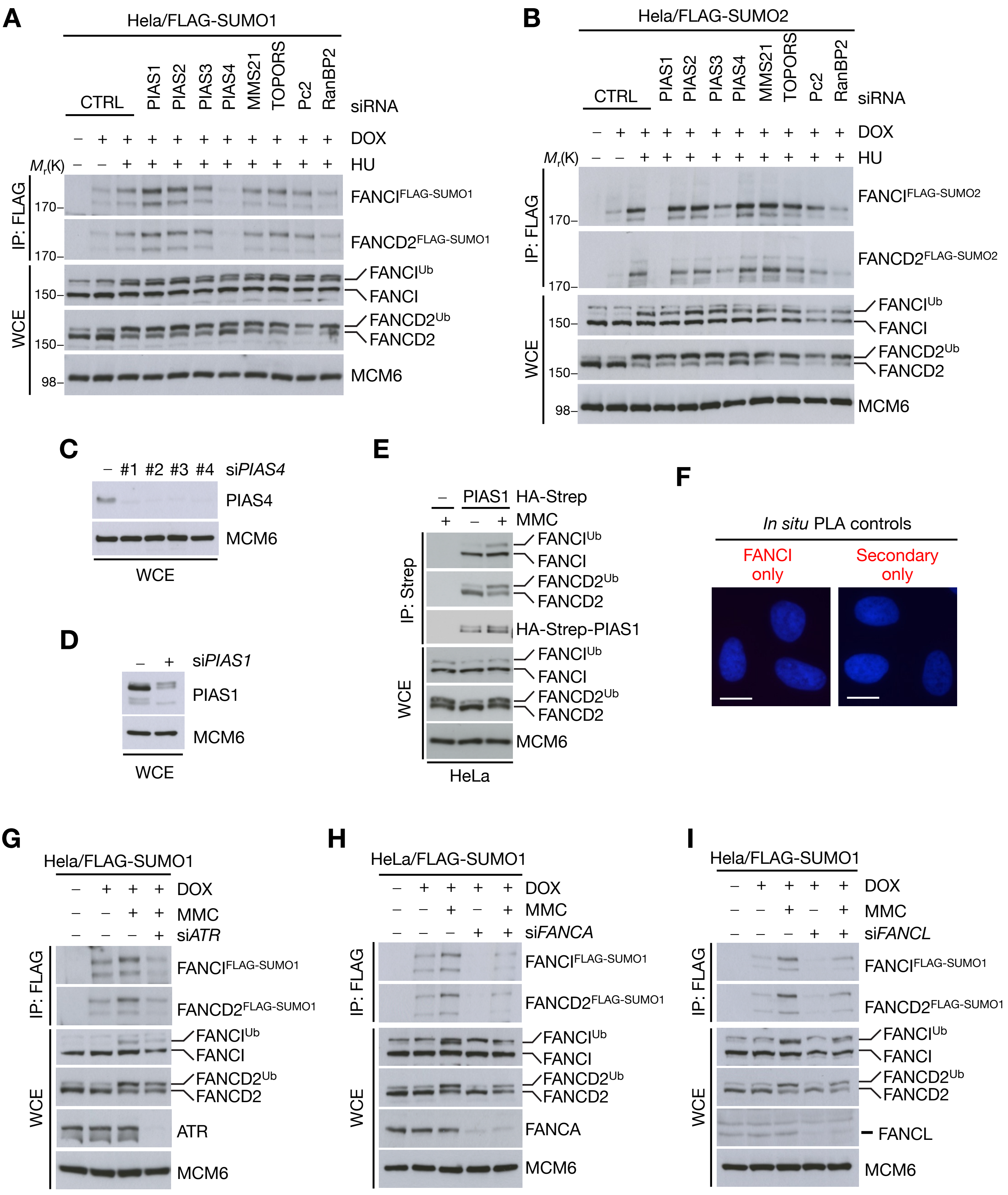

Figure S2 (related to Figure 2).
PIAS1, PIAS4, ATR and the FA core complex promote ID complex SUMOylation.

- **A.** HeLa/FLAG-SUMO1 cells were transfected with control (CTRL) or siRNAs against known SUMO E3 ligases, induced for FLAG-SUMO1 expression by DOX addition, then treated with HU (2 mM) for 24 h, lysed under denaturing conditions, immunopurified using FLAG beads and analysed by immunoblotting using the indicated antibodies.
- **B.** Same as (A) except for SUMO2.
- **C.** Depletion efficiencies of PIAS4 siRNAs used in this study. PIAS4(#4) was typically used in most experiments.
- **D.** Depletion efficiencies of PIAS1 siRNA used in this study.
- **E.** U2OS cells were transfected with HA-Strep-PIAS1 or empty vector (–) for 24 h and then subjected to MMC treatment (1 μM) for a further 24 h. HA-Strep-PIAS1 complexes were purified with Strep-Tactin Sepharose and analysed by immunoblotting with the indicated antibodies.
- **F.** Antibody controls related to the *in situ* promixity ligation assay shown in Fig. 2E, either lacking primary antibody or secondary antibody. Scale bar, 10 μm.
- **G.** HeLa/FLAG-SUMO1 cells were transfected with control or ATR siRNA, induced for FLAG-SUMO1 expression by DOX addition, treated with MMC (1 μM) for 24 h, then analysed as in (A).
- **H.** Same as above but with FANCA siRNA transfection.
- **I.** Same as above but with FANCL siRNA transfection.

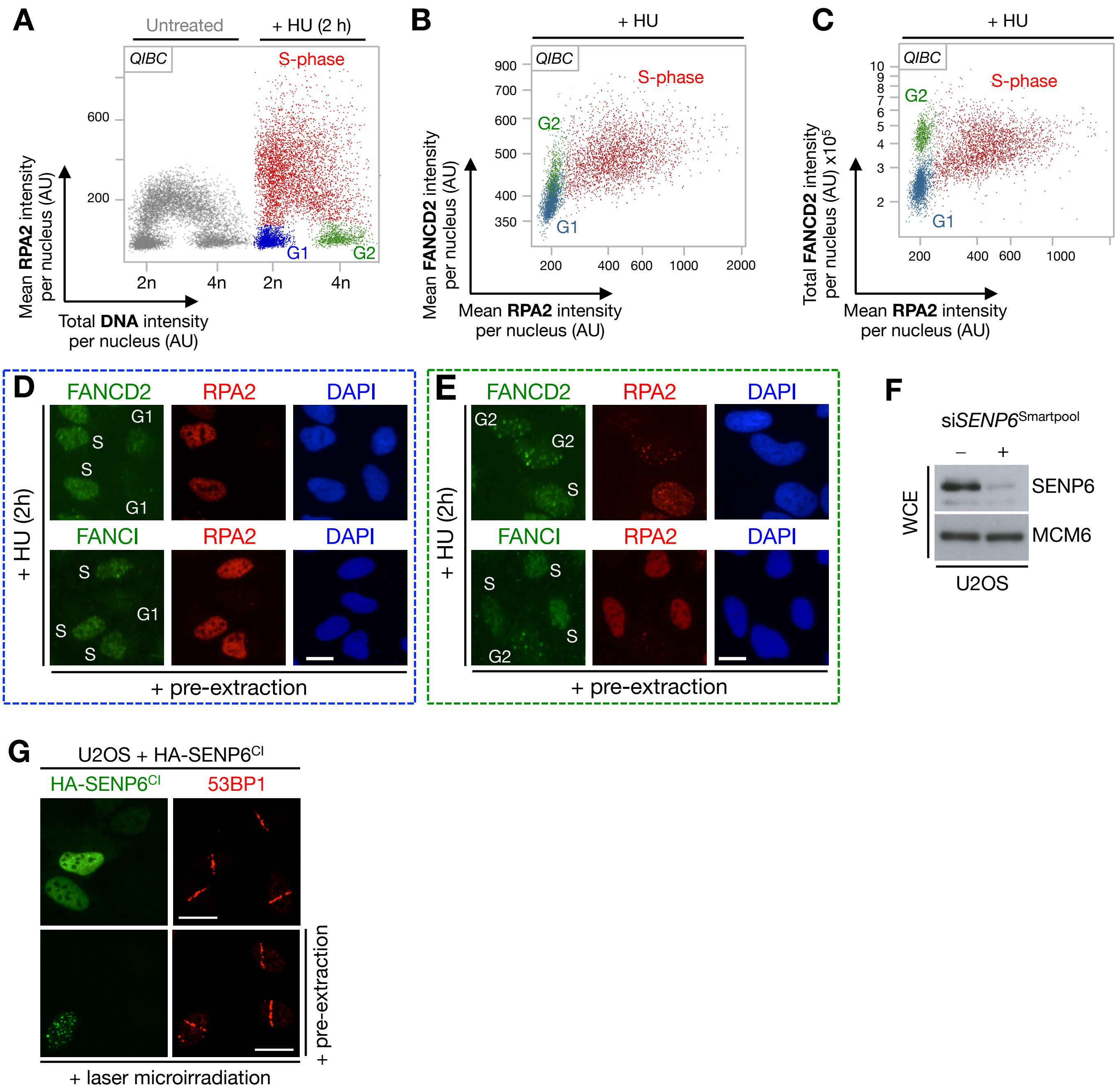
A
Untreated
+ HU (2 h)
QIBC
S-phase
G1
G2
600
200
2n
4n
2n
4n
Mean RPA2 intensity per nucleus (AU)
Total DNA intensity per nucleus (AU)
B
+ HU
QIBC
S-phase
G2
G1
900
700
600
500
400
350
200
400
600
1000
2000
Mean FANCD2 intensity per nucleus (AU)
Mean RPA2 intensity per nucleus (AU)
C
+ HU
QIBC
S-phase
G2
G1
10
9
8
7
6
5
4
3
2
200
400
600
1000
Total FANCD2 intensity per nucleus (AU) x10^5
Mean RPA2 intensity per nucleus (AU)
D
FANCD2
RPA2
DAPI
FANCI
+ HU (2h)
G1
S
+ pre-extraction
E
FANCD2
RPA2
DAPI
FANCI
+ HU (2h)
G2
S
+ pre-extraction
F
siSENP6Smartpool
–
+
WCE
SENP6
MCM6
U2OS
G
U2OS + HA-SENP6CI
HA-SENP6CI
53BP1
+ pre-extraction
+ laser microirradiation

Figure S3 (related to Figure 3).
Analysis of ID complex chromatin dynamics after replication stress using QIBC.

- **A.** U2OS cells were treated with HU (2 mM) for 2 h, pre-extracted *in situ* with detergent, fixed and then immunostained with FANCD2 and RPA2 antibodies together with DAPI to stain nuclear DNA content. QIBC can be used to discriminate RPA that is dynamically loaded on ssDNA produced by HU-induced fork stalling, compare left and right (Toledo et al., 2013).
- **B.** As in (A) except the mean FANCD2 intensity is plotted against mean RPA2 intensity. Previous assessment of S-phase cells in (A) allows discrimination of S-phase chromatin loading of FANCD2 (red).
- **C.** As in (B) except total FANCD2 intensity is plotted against mean RPA2 intensity. Note that FANCD2 exists in three cellular populations after isolation of chromatin bound proteins using *in situ* pre-extraction: 1. low RPA, low FANCD2 (G1-phase); 2. low RPA, high FANCD2 (G2-phase telomere-associated structures (Fan et al., 2009); 3. intermediate-to-high FANCD2, high RPA (S-phase). FANCI exhibits exactly the same dynamics (data not shown).
- **D.** Examples of images obtained from the high-content microscope used to generate data in A-C, showing predominantly G1 and S-phase cells.
- **E.** As in (D), but showing mainly S and G2 cells.
- **F.** Assessment of siSENP6^{Smartpool} depletion efficiency by immunoblotting.
- **G.** U2OS were transfected with HA-Strep-SENP6^{CI}, subjected to laser microirradiation, pre-extracted or not, fixed and immunostained with the indicated antibodies. Scale bar, 10 µm.

Gibbs-Seymour et al., Figure S4

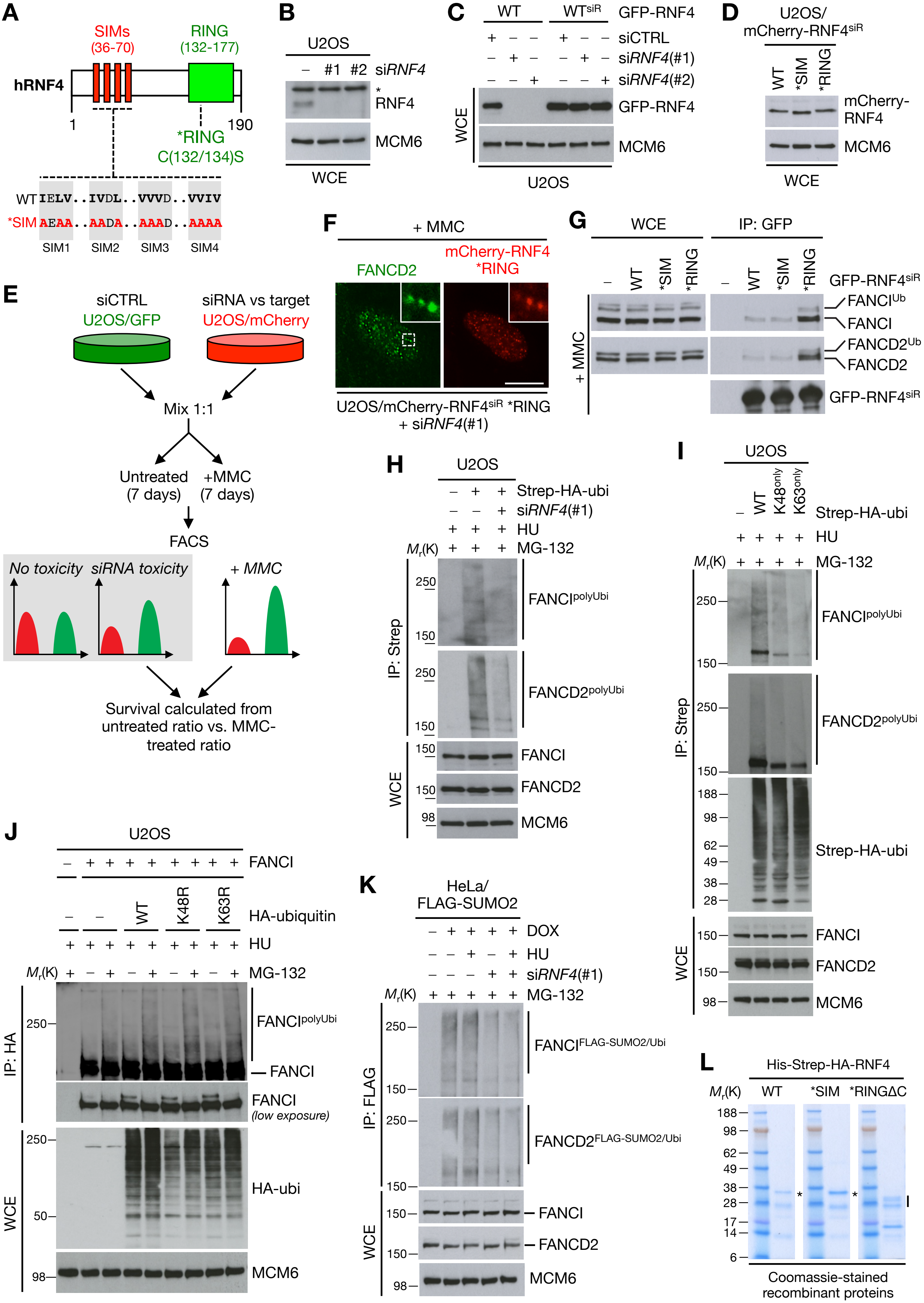

Figure S4 (related to Figure 4).
RNF4-mediated polyubiquitylation of the ID complex.

- **A.** RNF4 mutants used in this study. The hydrophobic residues of the N-terminal SIM region were mutated to alanines to give rise to the *SIM mutant. Two cysteines in the C-terminal RING domain were mutated to serine, to create the *RING mutant.
- **B.** Immunoblot assessment of RNF4 siRNAs used in this study.
- **C.** U2OS cells were transfected with control (CTRL) or RNF4 siRNA then transfected with RNF4 or RNF4 siRNA-resistant ($RNF4^{siR}$) expression constructs, before whole cell extracts were assessed by immunoblotting to determine efficiency of $RNF4^{siR}$ resistance to RNF4 siRNAs.
- **D.** Generation of U2OS stable cell lines expressing similar levels of mCherry-$RNF4^{siR}$ wild type and mutant alleles, as assessed by immunoblotting with the indicated antibodies.
- **E.** Schematic showing the principle of the multicolor competition assay (MCA) (Smogorzewska et al., 2007) used in Fig 4A.
- **F.** The U2OS/mCherry-$RNF4^{siR}$ *RING mutant was transfected with RNF4 siRNA, treated with MMC (0.3 µM) for 24 h, pre-extracted, fixed and immunostained with FANCD2 antibody. Scale bar, 10 µm.
- **G.** U2OS cells were transfected with GFP-$RNF4^{siR}$ constructs, treated with MMC (1 µM) for 4 h, before immunoprecipitation with GFP-Trap beads followed by immunoblotting with the indicated antibodies.
- **H.** U2OS/HA-Strep-ubiquitin cells were transfected with control or RNF4 siRNA, treated with HU (2 mM) for 24 h and in the last 4 h were treated with the proteasome inhibitor MG132 (10 µM), before cells were lysed under denaturing conditions, immunopurified using Strep-Tactin Sepharose and analysed by immunoblotting with indicated antibodies.
- **I.** U2OS cells were transfected with wild type HA-Strep-ubiquitin (WT), or two derivatives containing only one available lysine, K48 ($K48^{only}$) or K63 ($K63^{only}$), exposed to HU (2 mM) for 24 h and in the last 6 h were treated with the proteasome inhibitor MG132 (10 µM), before cells were lysed under denaturing conditions, immunopurified using Strep-Tactin Sepharose and analysed by immunoblotting with the indicated antibodies.
- **J.** U2OS/FANCI cells were transfected with wild type HA-Strep-ubiquitin (WT), or two derivatives containing K48R or K63R mutations, treated with HU (2 mM) for 24 h and in the last 4 h were treated with the proteasome inhibitor MG132. Ubiquitin-associated FANCI was then analysed after HA purification and immunoblotting with antibody against endogenous FANCI.
- **K.** HeLa/FLAG-SUMO2 cells were transfected with control or RNF4 siRNA, induced for SUMO expression by DOX addition, then treated with HU (2 mM) for 24 h and in the last 6 h treated with MG132, then lysed under denaturing conditions, immunopurified using FLAG beads and analysed by immunoblotting using the indicated antibodies.
- **L.** Coomassie-stained gels of recombinant His-Strep-HA-RNF4 and UBCH5b proteins used in this study.

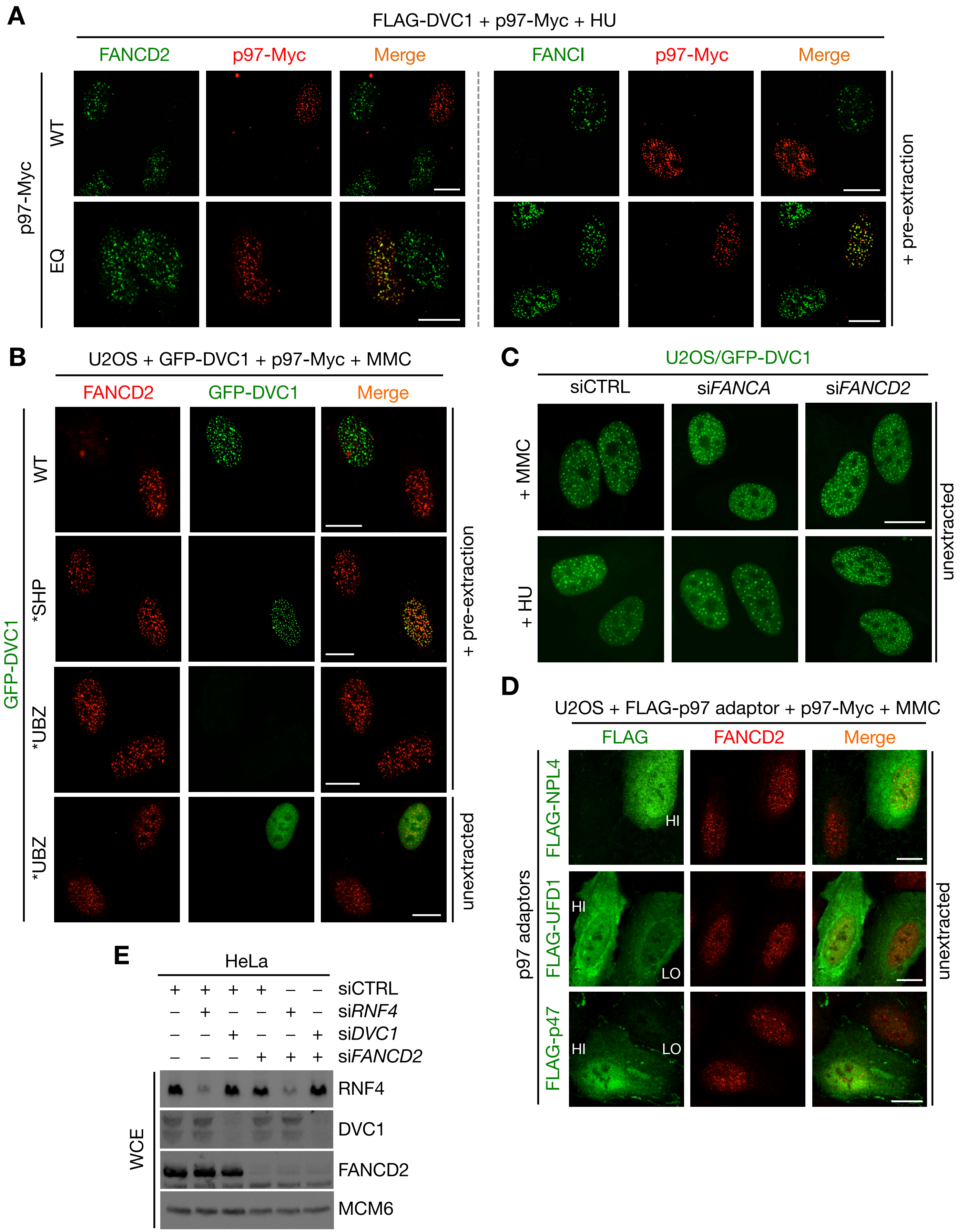
A
FLAG-DVC1 + p97-Myc + HU
FANCD2
p97-Myc
Merge
FANCI
p97-Myc
Merge
p97-Myc
WT
EQ
+ pre-extraction
B
U2OS + GFP-DVC1 + p97-Myc + MMC
FANCD2
GFP-DVC1
Merge
GFP-DVC1
WT
*SHP
*UBZ
*UBZ
+ pre-extraction
unextracted
C
U2OS/GFP-DVC1
siCTRL
siFANCA
siFANCD2
+ MMC
+ HU
unextracted
D
U2OS + FLAG-p97 adaptor + p97-Myc + MMC
FLAG
FANCD2
Merge
p97 adaptors
FLAG-NPL4
FLAG-UFD1
FLAG-p47
HI
LO
unextracted
E
HeLa
siCTRL
siRNF4
siDVC1
siFANCD2
WCE
RNF4
DVC1
FANCD2
MCM6

Figure S5 (related to Figure 5).
DVC1 is the specific p97 adaptor that promotes extraction of the ID complex in a ubiquitin-dependent manner.

- **A.** U2OS cells were co-transfected with FLAG-DVC1 and p97-Myc wild type (WT) or ATPase-dead E578Q (EQ), treated with HU (2 mM) for 24 h and then pre-extracted, fixed and immunostained with the indicated antibodies. Scale bar, 10 µm.
- **B.** U2OS cells were co-transfected with GFP-DVC1 wild type (WT), *SHP or *UBZ together and p97-Myc, treated with MMC (0.3 µM) for 24 h and then pre-extracted or not, fixed and immunostained with the indicated antibodies. Note that the GFP-DVC1 ubiquitin-binding deficient *UBZ mutant is completely solubilised followed pre-extraction, indicating that it is not stably retained on chromatin in response to replication stress (unextracted panel). Scale bar, 10 µm.
- **C.** U2OS/GFP-DVC1 cells were transfected with CTRL, FANCA or FANCD2 siRNA, treated with either HU (2 mM) or MMC (0.3 µM) for 24 h and then fixed. Scale bar, 10 µm.
- **D.** U2OS cells were co-transfected with FLAG-NPL4, FLAG-UFD1 or FLAG-p47 together with p97-Myc, treated with MMC (0.3 µM) for 24 h and then fixed and immunostained with the indicated antibodies. Note that each of these p97 adaptor proteins was unable to promote extraction of the ID complex after ICL formation. Furthermore, none were resistant to an *in situ* pre-extraction step (data not shown), indicating that they are not stably recruited to stalled replication forks. High (HI) and low (LO) expressing cells were included for comparison. Scale bar, 10 µm.
- **E.** Immunoblot analysis of HeLa cells used for clonogenic assays in Fig 5E.

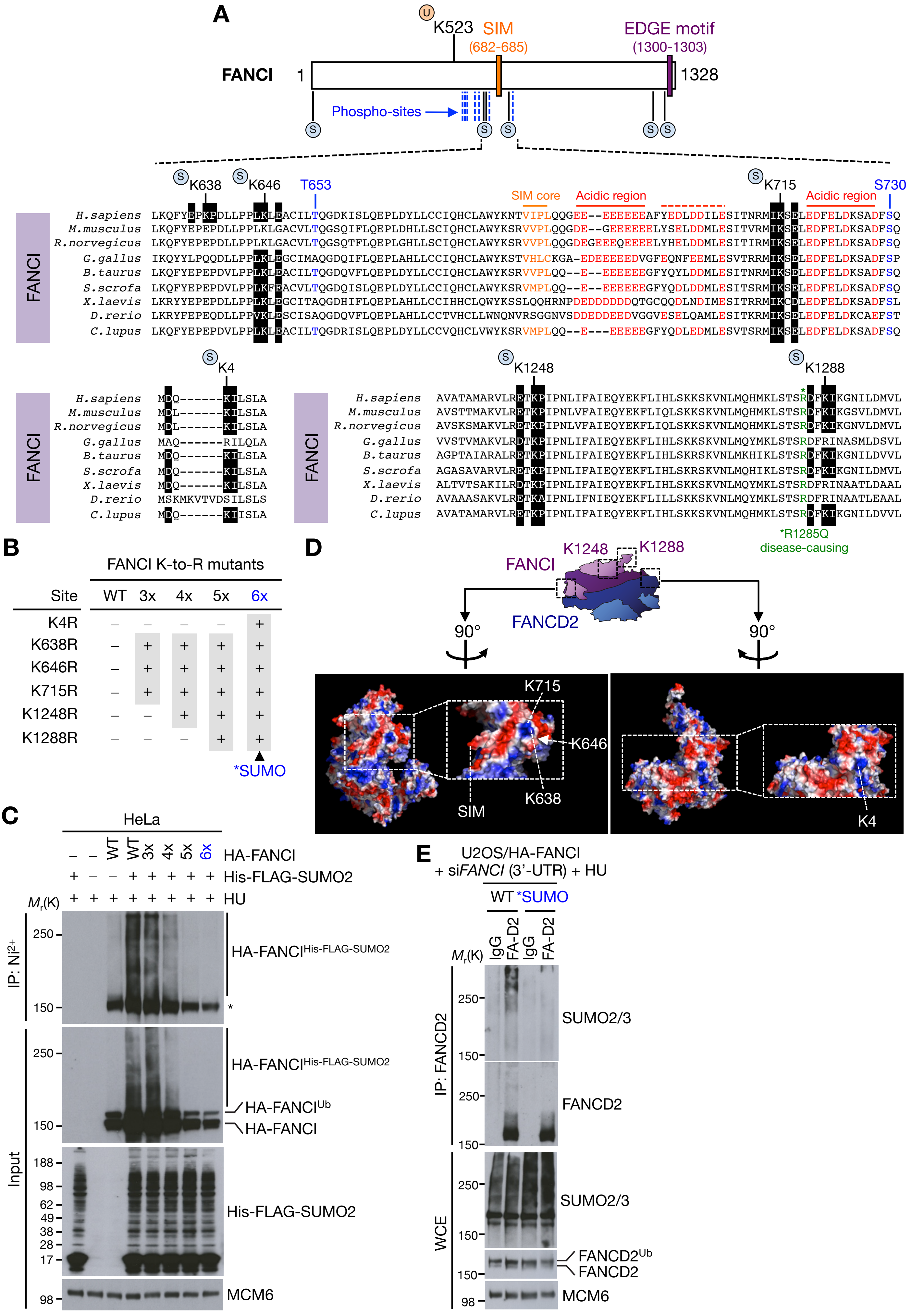
A
FANCI
1
1328
K523
SIM
(682-685)
EDGE motif
(1300-1303)
Phospho-sites
K638
K646
T653
SIM core
Acidic region
K715
Acidic region
S730
H.sapiens
M.musculus
R.norvegicus
G.gallus
B.taurus
S.scrofa
X.laevis
D.rerio
C.lupus
K4
K1248
K1288
*R1285Q
disease-causing
B
FANCI K-to-R mutants
Site
WT
3x
4x
5x
6x
K4R
K638R
K646R
K715R
K1248R
K1288R
*SUMO
C
HeLa
HA-FANCI
His-FLAG-SUMO2
HU
Mr(K)
IP: Ni2+
Input
HA-FANCIHis-FLAG-SUMO2
HA-FANCIUb
HA-FANCI
His-FLAG-SUMO2
MCM6
D
FANCI
FANCD2
90°
K715
K646
K638
SIM
K4
E
U2OS/HA-FANCI
+ siFANCI (3'-UTR) + HU
WT *SUMO
IgG
FA-D2
IP: FANCD2
WCE
SUMO2/3
FANCD2
FANCD2Ub
MCM6

Figure S6 (related to Figure 6).
FANCI SUMOylation regulates ID complex chromatin retention.

- **A.** Location and protein sequence alignment of potential SUMO sites in FANCI.
- **B.** FANCI SUMO site K-to-R expression constructs used in this study. The HA-FANCI *SUMO mutant contains all six K-to-R substitutions.
- **C.** U2OS cells were transfected with HA-FANCI wild type (WT) or SUMO site mutants and where indicated His-FLAG-SUMO2, subjected to HU treatment (2 mM) and 24 h later SUMO conjugates were purified under denaturing conditions using Ni^{2+} agarose. SUMO-modified FANCI was analysed by immunoblotting with HA antibody. *, denotes unmodified FANCI in immunoprecipitate.
- **D.** FANCI SUMO sites in the context of the previously described FANCD2/FANCI crystal structure (Joo et al., 2011) (PDB: 3S4W). Electrostatic surface and images were created using Pymol. Notably, the K715 residue is followed by a string of conserved negatively charged amino acids and an SQ phosphorylation site, which further classifies the K715 site as a negatively charged amino acid dependent SUMOylation motif (NDSM) (Yang et al., 2006). The negatively charged amino acids within this specialised SUMO motif promotes interaction with a basic patch on the SUMO E2 UBC9, thus targeting UBC9 to substrates to promote their SUMOylation (Mohideen et al., 2009). Viewed within the context of the ID complex crystal structure, the FANCI K4, K638, K715, K1248 and K1288 are all surface accessible for the SUMOylation machinery. In contrast, the K646 residue is buried within the ID complex and would require restructuring of the complex in order to undergo SUMOylation.
- **E.** U2OS/HA-FANCI WT or *SUMO cells transfected with FANCI siRNA against the 3'-UTR were treated with HU (2 mM) for 24 h and lysed under denaturing conditions before immunoprecipitation with anti-FANCD2 antibody or pre-immune serum (IgG). Bound material was analysed by immunoblotting with the indicated antibodies.

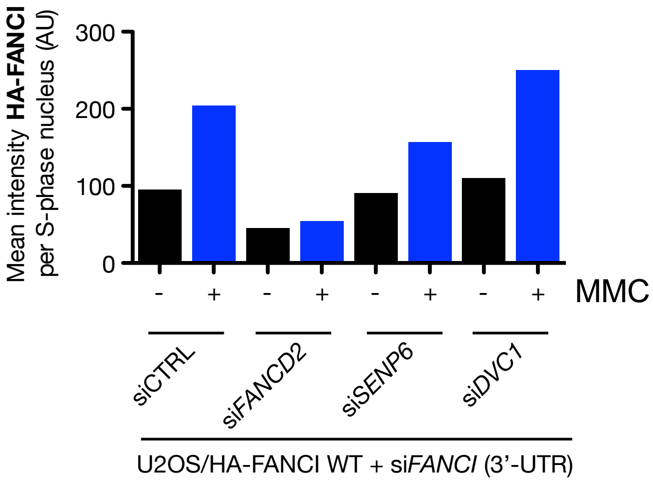
Mean intensity HA-FANCI per S-phase nucleus (AU)
300
200
100
0
- + - + - + - +
MMC
siCTRL
siFANCD2
siSENP6
siDVC1
U2OS/HA-FANCI WT + siFANCI (3'-UTR)

Figure S7 (related to Figure 7).
HA-FANCI WT phenocopies endogenous FANCI.
U2OS/HA-FANCI WT cells were depleted of endogenous FANCI together with FANCD2, SENP6, RNF4 or DVC1 and processed for QIBC. Results of a representative experiment are shown.

Supplemental Experimental Procedures

Plasmids and siRNA

Expression plasmids encoding His-FLAG-SUMO2, HA-Strep-PIAS1, HA-Strep-PIAS4, Myc-p97, FLAG-NPL4, FLAG-p47 and FLAG-UFD1 were described previously (Danielsen et al., 2012; Mosbech et al., 2012). A plasmid expressing pcDNA4/TO-HA-Strep-SENP6 was generated using established protocols. A plasmid expressing human HA-FANCI was a kind gift from Tony Huang (NYU Medical School, USA) and was subcloned into peGFP-C1 (Clontech). pcDNA3/HA-ubiquitin WT, K48R and K63R were described previously together with the pcDNA4/TO-HA-Strep-ubiquitin WT, K48-only and K63-only plasmids (Damgaard et al., 2012). RNF4 WT and *SIM cDNAs were synthesised by Eurofins MWG and both were rendered insensitive to two independent non-overlapping siRNAs by silent mutation of the following underlined nucleotides: siRNF4(#1) target – GAATGGACGTCTCATCGTT; siRNF4(#2) target – GACAGAGACGTATATGTGA. The RNF4 WT and *SIM cDNAs were cloned into pAcGFP-C1 and pmCherry-C1 expression vectors (both Clontech), to produce siRNA-Resistant GFP-RNF4^{siR} and mCherry-RNF4^{siR} WT and *SIM constructs. GFP-RNF4^{siR} and mCherry-RNF4^{siR} *RING mutant (C132S/C134S) was produced by site-directed mutagenesis. Cloning and site-directed mutagenesis was performed with PrimeSTAR Max Polymerase (Clontech) and KOD Hot Start Polymerase (Novagen). All constructs were verified by sequencing. Primer sequences are available upon request. Plasmid transfections were carried out using either GeneJuice (Novagen) or Fugene 6 (Promega) according to the manufacturer's instructions. Transfection of siRNAs was performed with RNAiMAX (Invitrogen) according to the manufacturer's instructions. The siRNA sequences and associated references used in this study were:
ATR (5'-CCUCCGUGAUGUUGCUUGA-3') (Casper et al., 2004),
control (CTRL) (5'-GGGAUACCUAGACGUUCUA-3') (Mosbech et al., 2012),
FANCA (5'-GCAGGUCACGGUUGAUGUA-3') (Liu et al., 2010),
FANCD2 (5'-CAACAUACCUCGACUCAUU-3') (Liu et al., 2010),
FANCI (5'-GCAGAAAGAAAUAGCGUCU-3') (Liu et al., 2010),
FANCI (5'-UTR) (5'-GGAAGUUUGUGGCGGAGUU-3') (this study), FANCI (3'-UTR) (5'-GCGCUUCACCUGAAAGAUA-3') (this study), FANCL (5'-GACAAGAGCUGUAUGCACU-3') (Meetei et al., 2003)
FANCM (5'-AGACAUCGCUGAAUUUAAA -3') (Xue et al., 2008),
MMS21 (5'-CUCUGGUAUGGACACACAGCU-3') (Galanty et al., 2009),
Pc2(#1) (5'-CGUGGGAACCGGAGGAGAA-3'),
Pc2(#2) (5'-GUUUGUACGUGGUGUUAUU-3'),
PIAS1 (5'-CGAAUGAACUUGGCAGAAA-3') (Galanty et al., 2009),
PIAS2 (5'-CUUGAAUAUUACAUCUUUA-3') (Galanty et al., 2009),
PIAS3 (5'-CCCUGAUGUCACCAUGAAA-3') (Galanty et al., 2009),
PIAS4(#1) (5'-GGAGUAAGAGUGGACUGAA-3') (Galanty et al., 2009),
PIAS4(#2) (5'-AGGCACUGGUCAAGGAGAA-3') (Galanty et al., 2009),
PIAS4(#3) (5'-AGCUGCCGUUCUUUAAUAU-3') (this study),
PIAS4 (#4) (5'-CAAGACAGGUGGAGUUGAU-3') (this study),

RNF4(#1) (5’-GAAUGGACGUCUCAUCGUU-3’) (Galanty et al., 2012; Yin et al., 2012),
RNF4(#2) (5’-GACAGAGACGUAUAUCUGA-3’) (Galanty et al., 2012; Yin et al., 2012),
RNF111 (5’-GGAUAUUAAUGCAGAGGAA-3’) (Poulsen et al., 2013),
RanBP2 (5’-GGACAGUGGGAUUGUAGUG-3’) (Joseph et al., 2004),
SENP1 (siGENOME Smartpool # M-006357-00), SENP2 (siGENOME Smartpool #M-006033-01), SENP3 (siGENOME Smartpool #M-006034-01), SENP5 (siGENOME Smartpool #M-005946-01), SENP6 (siGENOME Smartpool #M-006044-01), SENP7 (siGENOME Smartpool #M-006035-01),
SENP6 (5’-GAAAGUGAAGGAGAUACAG-3’),
TOPORS (5’-CAAGGAGCCUGUCUAGUAA-3’).

Cell culture

Unless otherwise indicated, the following doses of genotoxic agents were used: Mitomycin C (MMC, 1 µM), Hydroxyurea (HU, 2 mM), Aphidicolin (APH, 4 µM), IR (10 Gy), Cisplatin (5 µM) and UV (20 J/m^2).

Purification and detection of endogenously SUMOylated proteins

Purification of endogenous FANCI or FANCD2 for SUMO2/3 analysis was carried out essentially as described (Barysch et al., 2014). Briefly, cells were lysed in the presence of 1% SDS, sonicated, boiled at 95°C for 10 minutes and then diluted 1:10 in RIPA dilution buffer. Lysates were passed through a 0.45µm filter and incubated with 2 µg of FANCI or FANCD2 antibody or control IgG overnight at 4°C. Protein A sepharose was then added for 4 h and bound material was subsequently washed with RIPA buffer containing 0.1% wt/vol SDS. Bound proteins were eluted with sample buffer.

Antibodies

Antibodies used in this study included: mouse monoclonals to SUMO2/3 (Abcam), mCherry, 6xHis (Clontech), RPA2* (clone 9H8, Diagnostic Biosystems), γH2AX* (Millipore), GFP, HA*, (Santa Cruz), FLAG, vinculin (Sigma); rabbit monoclonals/polyclonals to γH2AX*, FANCD2*, PIAS1, RPA1* (clone EPR3472), RPA2* (clone EPR2877Y), SUMO2/3 (Abcam), FANCA, FANCI*, SENP6 (Bethyl Laboratories), Chk1(pS317), Histone H2AX, NF-κB, PIAS4 (Cell Signaling), FANCD2* (Novus Biologicals), RNF4 (a kind gift from J. Palvimo, University of Eastern Finland, Kuopio, Finland), FANCL (a kind gift from Weidong Wang, National Institute of Aging, USA); goat polyclonals to ATR, DVC1 and MCM6 (Santa Cruz); rat monoclonal to HA* (Sigma). *, denotes that the antibody was used for QIBC.

In vitro SUMOylation and STUbL assays

For *in vitro* SUMOylation assays, components were added to a total reaction volume of 30 µl in SUMOylation buffer (50 mM Tris, pH 7.5; 5 mM $MgCl_2$; 0.6 mM DTT; 2 mM NaF) as follows: E1 - SAE1/2 (Boston Biochem) - 30 µM, E2 - UBC9 (Boston Biochem) – 167 µM, E3 - PIAS1 (Enzo Life Sciences) or PIAS4 (this study) – 120 µM, 15 mM SUMO1 or SUMO2 (both Boston Biochem) – 667 nM FANCI or FANCD2. Reactions were incubated at 30°C for 2 h and

stopped by the addition of 4x SDS-PAGE loading buffer. For STUbL assays, reactions were scaled up 3-fold. After the *in vitro* SUMOylation assay, extracts were diluted in 500 µl binding buffer (20mM Tris, pH 7.5; 150 mM NaCl; 0.05% NP-40; 1 mM imidazole) and added to 15 µl (packed volume) Ni^{2+} agarose for 2 h at 4°C. Bound proteins were washed extensively in 50 mM Tris pH 7.5 and subjected to an *in vitro* ubiquitylation assay, using the following components: E1 (Boston Biochem) – 28 µM, E2 – UBCH5c (Boston Biochem) – 400 µM , E3 - RNF4 (this study) – 400 µM, Myc-ubiquitin (Boston Biochem) – 18 mM. Beads were incubated at 37°C for 90 mins with shaking and then washed extensively before the addition of SDS-PAGE loading buffer containing 250 mM imidazole. Reaction products were analysed by immunoblotting.

QIBC

Quantitative image-based cytometry (QIBC) was performed exactly as described (Toledo et al., 2013). Briefly, cells were pre-extracted, fixed and stained as described above before nuclear DNA was stained with DAPI (Molecular Probes) for 4 min in 0.01% Tween-20/PBS. Cells were mounted in Mowiol 488 medium (EMD Millipore). Images were acquired with an Olympus IX-81 wide-field microscope equipped with an MT20 Illumination system and a digital monochrome Mahatsu C9100 CCD camera. Olympus UPLSAPO 10x/0.4 NA, 20x/0.75 NA and 40x/0.95 NA objectives were used. Automated and unbiased image analysis was carried out with the ScanR acquisition software. In experiments using the 10x objective, 4,000-10,000 cells were analysed per datapoint. Data was exported and processed in Spotfire (Tibco) software.

Mass spectrometry-based analysis of FANCI/FANCD2 SUMO sites

To determine FANCD2 and FANCI SUMO2 sites by mass spectrometry, *in vitro* SUMOylation assays were carried out as described above except with recombinant human SUMO2 (Q87R), a kind gift from Dr Alfred Vertegaal (Leiden University Medical Center, the Netherlands). *In vitro* SUMOylation reactions were performed for 2 h at 37°C, stopped by boiling in SDS-PAGE loading buffer and were separated by SDS-PAGE using a 4-12% NuPAGE gel (Life Technologies). Peptides were recovered from the SDS-PAGE gel using a standard in-gel digestion protocol (Lundby and Olsen, 2011). Peptide fractions were analyzed by online nanoflow LC-MS/MS using a Proxeon easy nLC 1000 system connected to an Q-Exactive mass spectrometer (Thermo Scientific), as described (Kelstrup et al., 2012). Raw data was computationally processed using MaxQuant (developer version 1.4.0.3) and searched against the UniProt database (April 2012 release) using the integrated Andromeda search engine (http://www.maxquant.org) (Cox and Mann, 2008; Cox et al., 2011). The SUMO remnant peptide (QQTGG) derived from tryptic cleavage of Q87R mutant SUMO was included as a variable modification on lysine residues using the default settings in MaxQuant.

Supplemental References

Barysch, S.V., Dittner, C., Flotho, A., Becker, J., and Melchior, F. (2014). Identification and analysis of endogenous SUMO1 and SUMO2/3 targets in mammalian cells and tissues using monoclonal antibodies. Nature protocols *9*, 896-909.

Casper, A.M., Durkin, S.G., Arlt, M.F., and Glover, T.W. (2004). Chromosomal instability at common fragile sites in Seckel syndrome. American journal of human genetics *75*, 654-660.

Cox, J., and Mann, M. (2008). MaxQuant enables high peptide identification rates, individualized p.p.b.-range mass accuracies and proteome-wide protein quantification. Nature biotechnology *26*, 1367-1372.

Cox, J., Neuhauser, N., Michalski, A., Scheltema, R.A., Olsen, J.V., and Mann, M. (2011). Andromeda: a peptide search engine integrated into the MaxQuant environment. Journal of proteome research *10*, 1794-1805.

Damgaard, R.B., Nachbur, U., Yabal, M., Wong, W.W., Fiil, B.K., Kastirr, M., Rieser, E., Rickard, J.A., Bankovacki, A., Peschel, C.*, et al.* (2012). The ubiquitin ligase XIAP recruits LUBAC for NOD2 signaling in inflammation and innate immunity. Molecular cell *46*, 746-758.

Danielsen, J.R., Povlsen, L.K., Villumsen, B.H., Streicher, W., Nilsson, J., Wikstrom, M., Bekker-Jensen, S., and Mailand, N. (2012). DNA damage-inducible SUMOylation of HERC2 promotes RNF8 binding via a novel SUMO-binding Zinc finger. The Journal of cell biology *197*, 179-187.

Fan, Q., Zhang, F., Barrett, B., Ren, K., and Andreassen, P.R. (2009). A role for monoubiquitinated FANCD2 at telomeres in ALT cells. Nucleic acids research *37*, 1740-1754.

Galanty, Y., Belotserkovskaya, R., Coates, J., and Jackson, S.P. (2012). RNF4, a SUMO-targeted ubiquitin E3 ligase, promotes DNA double-strand break repair. Genes & development *26*, 1179-1195.

Galanty, Y., Belotserkovskaya, R., Coates, J., Polo, S., Miller, K.M., and Jackson, S.P. (2009). Mammalian SUMO E3-ligases PIAS1 and PIAS4 promote responses to DNA double-strand breaks. Nature *462*, 935-939.

Joo, W., Xu, G., Persky, N.S., Smogorzewska, A., Rudge, D.G., Buzovetsky, O., Elledge, S.J., and Pavletich, N.P. (2011). Structure of the FANCI-FANCD2 complex: insights into the Fanconi anemia DNA repair pathway. Science *333*, 312-316.

Joseph, J., Liu, S.T., Jablonski, S.A., Yen, T.J., and Dasso, M. (2004). The RanGAP1-RanBP2 complex is essential for microtubule-kinetochore interactions in vivo. Current biology : CB *14*, 611-617.

Kelstrup, C.D., Young, C., Lavallee, R., Nielsen, M.L., and Olsen, J.V. (2012). Optimized fast and sensitive acquisition methods for shotgun proteomics on a quadrupole orbitrap mass spectrometer. Journal of proteome research *11*, 3487-3497.

Knipscheer, P., Raschle, M., Smogorzewska, A., Enoiu, M., Ho, T.V., Scharer, O.D., Elledge, S.J., and Walter, J.C. (2009). The Fanconi anemia pathway promotes replication-dependent DNA interstrand cross-link repair. Science *326*, 1698-1701.

Liu, T., Ghosal, G., Yuan, J., Chen, J., and Huang, J. (2010). FAN1 acts with FANCI-FANCD2 to promote DNA interstrand cross-link repair. Science *329*, 693-696.
Long, D.T., Raschle, M., Joukov, V., and Walter, J.C. (2011). Mechanism of RAD51-dependent DNA interstrand cross-link repair. Science *333*, 84-87.
Lundby, A., and Olsen, J.V. (2011). GeLCMS for in-depth protein characterization and advanced analysis of proteomes. Methods Mol Biol *753*, 143-155.
Meetei, A.R., de Winter, J.P., Medhurst, A.L., Wallisch, M., Waisfisz, Q., van de Vrugt, H.J., Oostra, A.B., Yan, Z., Ling, C., Bishop, C.E.*, et al.* (2003). A novel ubiquitin ligase is deficient in Fanconi anemia. Nature genetics *35*, 165-170.
Mohideen, F., Capili, A.D., Bilimoria, P.M., Yamada, T., Bonni, A., and Lima, C.D. (2009). A molecular basis for phosphorylation-dependent SUMO conjugation by the E2 UBC9. Nature structural & molecular biology *16*, 945-952.
Mosbech, A., Gibbs-Seymour, I., Kagias, K., Thorslund, T., Beli, P., Povlsen, L., Nielsen, S.V., Smedegaard, S., Sedgwick, G., Lukas, C.*, et al.* (2012). DVC1 (C1orf124) is a DNA damage-targeting p97 adaptor that promotes ubiquitin-dependent responses to replication blocks. Nature structural & molecular biology *19*, 1084-1092.
Poulsen, S.L., Hansen, R.K., Wagner, S.A., van Cuijk, L., van Belle, G.J., Streicher, W., Wikstrom, M., Choudhary, C., Houtsmuller, A.B., Marteijn, J.A.*, et al.* (2013). RNF111/Arkadia is a SUMO-targeted ubiquitin ligase that facilitates the DNA damage response. The Journal of cell biology *201*, 797-807.
Raschle, M., Knipscheer, P., Enoiu, M., Angelov, T., Sun, J., Griffith, J.D., Ellenberger, T.E., Scharer, O.D., and Walter, J.C. (2008). Mechanism of replication-coupled DNA interstrand crosslink repair. Cell *134*, 969-980.
Smogorzewska, A., Matsuoka, S., Vinciguerra, P., McDonald, E.R., 3rd, Hurov, K.E., Luo, J., Ballif, B.A., Gygi, S.P., Hofmann, K., D'Andrea, A.D.*, et al.* (2007). Identification of the FANCI protein, a monoubiquitinated FANCD2 paralog required for DNA repair. Cell *129*, 289-301.
Toledo, L.I., Altmeyer, M., Rask, M.B., Lukas, C., Larsen, D.H., Povlsen, L.K., Bekker-Jensen, S., Mailand, N., Bartek, J., and Lukas, J. (2013). ATR Prohibits Replication Catastrophe by Preventing Global Exhaustion of RPA. Cell *155*, 1088-1103.
Xue, Y., Li, Y., Guo, R., Ling, C., and Wang, W. (2008). FANCM of the Fanconi anemia core complex is required for both monoubiquitination and DNA repair. Human molecular genetics *17*, 1641-1652.
Yang, S.H., Galanis, A., Witty, J., and Sharrocks, A.D. (2006). An extended consensus motif enhances the specificity of substrate modification by SUMO. The EMBO journal *25*, 5083-5093.
Yin, Y., Seifert, A., Chua, J.S., Maure, J.-F., Golebiowski, F., and Hay, R.T. (2012). SUMO-targeted ubiquitin E3 ligase RNF4 is required for the response of human cells to DNA damage. Genes & development *26*, 1196-1208.
Zhang, J., and Walter, J.C. (2014). Mechanism and regulation of incisions during DNA interstrand cross-link repair. DNA repair *19*, 135-142.
